# Dual targeting of TIGIT and VISTA in non-small-cell lung cancer immunotherapy

**DOI:** 10.17179/excli2025-8735

**Published:** 2025-08-28

**Authors:** Alaa A. A. Aljabali, Omar Gammoh, Esam Qnais, Abdelrahim Alqudah, Vijay Mishra, Yachana Mishra, Mohamed El-Tanani

**Affiliations:** 1 Department of Pharmaceutics and Pharmaceutical Technology, Faculty of Pharmacy, Yarmouk University, P.O. Box 566, Irbid, 21163, Jordan; 2 Department of Clinical Pharmacy and Pharmacy Practice, Faculty of Pharmacy, Yarmouk University, PO BOX 566, Irbid, 21163, Jordan; 3 Department of Biology and Biotechnology, Faculty of Science, The Hashemite University, Zarqa, Jordan; 4 Department of Clinical Pharmacy and Pharmacy Practice, Faculty of Pharmaceutical Sciences, The Hashemite University, Zarqa 13133, Jordan; 5 School of Pharmaceutical Sciences, Lovely Professional University, Phagwara (Punjab) 144411, India; 6 School of Bioengineering and Biosciences, Lovely Professional University, Phagwara (Punjab) 144411, India; 7 College of Pharmacy, Ras Al Khaimah Medical and Health Sciences University, Ras Al Khaimah, UAE

**Keywords:** non-small cell lung cancer, TIGIT, VISTA, dual immune checkpoint blockade, tumor microenvironment, T-cell exhaustion

## Abstract

This study investigated the therapeutic impact of dual immune checkpoint inhibition targeting TIGIT and VISTA in non-small cell lung cancer (NSCLC). Current monotherapies have failed to produce consistent and durable responses owing to tumor heterogeneity and immune evasion. By evaluating the biological and immunomodulatory roles of TIGIT and VISTA, this study provides a rationale for their simultaneous blockade. Preclinical models have shown that this dual strategy not only revitalizes T-cell function but also alters the suppressive tumor microenvironment, leading to improved antitumor immunity in mice. Preliminary clinical data suggest potential survival benefits; however, the long-term outcomes and resistance dynamics remain uncertain. These findings suggest a paradigm shift toward precision-designed, multi-target immunotherapies. Future studies should integrate molecular profiling, adaptive clinical trial designs, and follow-up models to optimize patient selection and sustain therapeutic benefits.

See also the graphical abstract(Fig. 1).

## Abbreviations

APCs: Antigen-presenting cells

CD4⁺ T cells: Cluster of differentiation 4-positive T lymphocytes

CD8⁺ T cells: Cluster of differentiation 8-positive T lymphocytes

CDS: Coding sequences

CMC: Chemistry, manufacturing, and controls

CTLA-4: Cytotoxic T-lymphocyte-associated protein 4

CXCL13: C-X-C motif chemokine ligand 13

DC: Dendritic cell

FDA: Food and Drug Administration

GITR: Glucocorticoid-induced tumor necrosis factor receptor-related protein

HPV: Human papillomavirus

ICIs: Immune checkpoint inhibitors

IFN-γ: Interferon gamma

IL-10: Interleukin-10

MDSCs: Myeloid-derived suppressor cells

mRNA: Messenger ribonucleic acid

NK cells: Natural killer cells

NSCLC: Non-small cell lung cancer

PD-1: Programmed cell death protein 1

PD-L1: Programmed death-ligand 1

RNA: Ribonucleic acid

SG7: Anti-VISTA monoclonal antibody (clone SG7)

TCR: T cell receptor

TIGIT: T cell immunoreceptor with Ig and ITIM domains

TILs: Tumor-infiltrating lymphocytes

TME: Tumor microenvironment

TNF-α: Tumor necrosis factor alpha

Tregs: Regulatory T cells

VISTA: V-domain Ig suppressor of T cell activation

## Introduction

Non-small cell lung cancer (NSCLC) is a serious global health challenge and one of the foremost causes of cancer-related deaths worldwide (Dorochowicz et al., 2024[37]; Tang et al., 2025[155]; Verma et al., 2024[161]). The stealthy way in which NSCLC is frequently diagnosed once it is advanced limits curative possibilities, emphasizing the need for innovative strategies. Conventional treatments, such as surgery, chemotherapy, and radiation, have long been the pillars of NSCLC management; however, their efficacy is frequently undermined by intrinsic or secondary resistance and considerable toxicity. Consequently, the need for more targeted and less toxic therapies has spurred remarkable advancements in immuno-oncology (Carmichael et al., 2018[18]; Fiste et al., 2024[48]; Marks et al., 2024[104]).

The arrival of immune checkpoint inhibitors (ICIs) has dramatically changed the treatment of NSCLC, with unprecedented gains in patient response rate and overall survival (Yan et al., 2024[179]). These drugs, including antibodies directed against programmed cell death protein 1 (PD-1), programmed death-ligand 1 (PD-L1), and cytotoxic T-lymphocyte-associated antigen 4 (CTLA-4), harness the power of the immune system to identify and destroy cancer cells. In contrast to autologous chemotherapy or radiation, which directly kill cancer cells, ICIs reanimate exhausted T cells and induce long-lasting anti-tumor immunity by blocking the inhibitory pathways that tumors hijack to escape immune detection (Liu et al., 2021[94]; Pandey et al., 2022[120]; Qing et al., 2022[133]; Yan et al., 2024[179]). This new way of fighting cancer has produced dramatic clinical gains in a subgroup of NSCLC patients and has even facilitated durable remission in advanced cases.

Despite such groundbreaking achievements, many patients with NSCLC still have suboptimal outcomes, highlighting the necessity for further investigation of new immune checkpoint approaches (Jeon et al., 2025[71]). Although the use of ICIs has enhanced survival, a noteworthy percentage of patients are not responsive to treatment or become resistant to it over time. This underlies the intricate interactions between the immune system, tumor, and tumor microenvironment; therefore, a sound understanding of immune escape and therapeutic resistance mechanisms is required. To answer the same, scientists are working energetically towards the identification of new immune checkpoint targets and combination regimens that are less encumbered by the limitations of existing treatments and enhance the outcomes for a wider cross-section of patients with NSCLC. The establishment of rational immune checkpoint combinations using innovative strategies is essential for the progression of the quest for the best therapy for NSCLC (Bilger et al., 2021[11]; Bronte et al., 2023[16]; Livanou et al., 2024[98]; Tostes et al., 2023[157]).

The therapeutic paradigm of NSCLC has seen a remarkable shift in recent years owing to dramatic developments in the field of immunotherapy. This shift represents a break from traditional treatment paradigms based on chemotherapy and radiotherapy to more innovative and targeted therapeutic approaches (Lazzari et al., 2023[82]). Of the numerous emerging therapeutic targets available today, immune checkpoint inhibitors have assumed a central role owing to their ability to modulate the intrinsic immune response of neoplastic cells. This novel therapeutic paradigm capitalizes on the body's immune apparatus to enlist immune cell action with greater efficacy to target malignancies (Lieber et al., 2024[89]). Central to the efficacy of such an approach are immune checkpoints, most significantly the PD-1/PD-L1 pathway; however, emerging and compelling evidence suggests that other immune checkpoints, including TIGIT and VISTA, may also be vital to the complex pathways of immune resistance and tumor evasion seen in NSCLC (Mariniello et al., 2025[103], Massafra et al., 2021[106]). 

**Figure 2:**
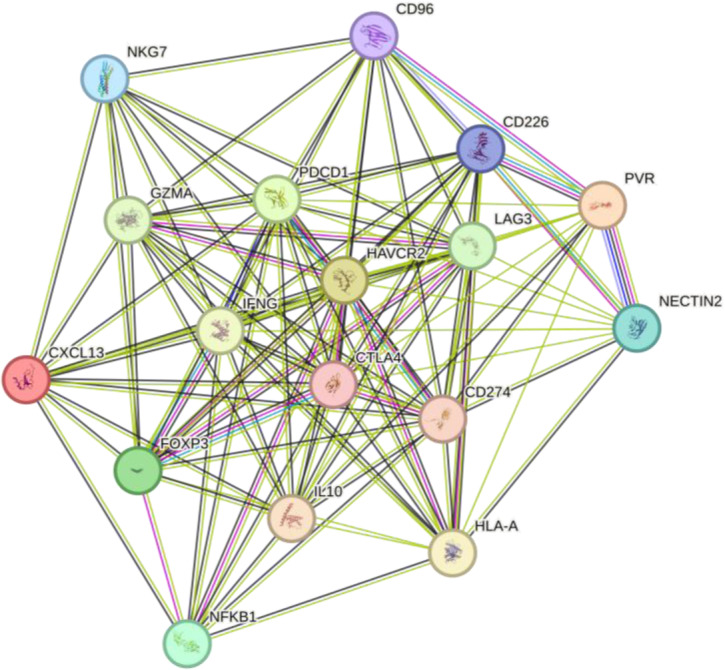
STRING protein-protein interaction network of immune checkpoint molecules relevant to NSCLC. The network highlights the functional associations among TIGIT, VISTA, CD226, CD274, PDCD1, CTLA4, and HAVCR2. Edges represent experimentally validated, co-expressed, and database-predicted interactions between the nodes. This visual framework supports the hypothesis that TIGIT and VISTA operate within a broader immunosuppressive axis that shapes the tumor microenvironment in non-small cell lung cancer.

Both TIGIT and VISTA are negative regulators of T cell function and have been identified as prospective contributory agents to the immunosuppressive tumor microenvironment characteristic of NSCLC (Figure 2(Fig. 2)). In particular, TIGIT is found on a heterogeneous population of immune cells, including T and NK cells, and interacts with ligands to decrease T-cell proliferation and cytokine production. Similarly, VISTA is found predominantly on myeloid cells and functions as both a ligand and a receptor, inhibiting T cell activation and facilitating an immunosuppressive microenvironment (Huang et al., 2022[67]; Massafra et al., 2021[106]; Zou et al., 2023[196]). The concept of rational targeting of both TIGIT and VISTA is a promising therapeutic modality aimed at bypassing the inhibitory signaling that affects immune responses against neoplastic cells. By targeting both checkpoints with inhibition, this therapeutic modality aims to boost T cell action and increase the immune-mediated assault on tumor tissues (Struckmeier et al., 2024[150]; Zhang et al., 2024[188]). 

Considering the intrinsic multifunctional reality of NSCLC and the varied redundant mechanisms used by the tumor to avoid immune surveillance, a therapeutic modality that includes targeted inhibition of both TIGIT and VISTA could represent a potent methodology to enhance overall therapeutic efficacy (Patel and Middleton, 2023[123]; Pescia et al., 2023[128]). This two-target strategy is based on the premise that simultaneous blockade of both pathways has the potential to induce synergistic effects and possibly overcome the problem of resistance commonly seen with monotherapy based on checkpoint inhibition. Broadening our understanding of the interplay between these two pathways and how their combined inhibition can repolarize the immune microenvironment promises to open new avenues for therapeutic intervention. Such groundbreaking studies could eventually set the stage for better patient outcomes in NSCLC (Chen et al., 2024[22]; De Giglio et al., 2021[31]). The significance of this initiative highlights the necessity for continued investigation, careful clinical testing, and the development of combined therapies with the potential to revolutionize the therapeutic paradigms of patients with lung cancer. This unfolding story in the management of NSCLC reflects the dynamism of the subject and its dedication to improving the lives of patients battling this devastating cancer (Chen et al., 2024[22]). As shown in Table 1(Tab. 1), the integration of TIGIT and VISTA checkpoint modulation through a precision dual-targeting strategy leads to significant improvements in immune activation potential and therapeutic efficacy, with projected response rates ranging from 25-68% (Kamali et al., 2023[76]).

## Background

NSCLC is the most prevalent type of lung cancer, accounting for approximately 85% of all diagnosed cases globally (Min, 2024[111]). Tumors are classified into several histological types, including adenocarcinoma, squamous cell carcinoma, and large-cell carcinoma. Despite innovations in early detection methods and therapeutic protocols, NSCLC remains the predominant cause of cancer-related deaths worldwide. The main causes are late-stage diagnosis and the intrinsic heterogeneity of the tumor itself, which pose serious challenges to tailoring therapeutic regimens to combat it (Alduais et al., 2023[1]; Kafková et al., 2024[75]; Min, 2024[111]). 

The immune system plays a central role in the identification and elimination of tumor cells; however, NSCLC has developed a complex system that evades immune perception and response. This highlights the pressing need for novel therapeutic modalities to address the complex challenges presented by NSCLC (Yoneda et al., 2018[182]). The last decade has seen a revolutionary effect of immunotherapy on the management of NSCLC. Among the multiplicity of available therapeutic options are checkpoint inhibitors, which have become the frontline warriors against cancer cell destruction. By selectively targeting a specific set of inhibitory checkpoint proteins, these agents induce robust T-cell-mediated attacks against cancer cells (Moya-Horno et al., 2018[116]; Wald, 2018[163]). Despite their efficiency in combating cancer cells, a considerable percentage of the population shows inherent or acquired resistance to immunotherapeutic agents, prompting extensive research into the biological mechanisms underlying such resistance (Frisone et al., 2022[50]). An interesting avenue of research is the combined targeting of different immunoregulatory proteins, which has been identified as a potentially useful therapeutic paradigm for overcoming the shortcomings of existing checkpoint-inhibitory therapy. 

One such protein functions as an immunosuppressive receptor on lymphocytes and plays a central role in controlling immune responses and modulating T-cell activation. The other protein is predominantly expressed in myeloid-derived cells and causes immunosuppressive activity in the tumor microenvironment (Cheever et al., 2022[20]; Liu et al., 2022[97]). Together, these targets offer a promising platform for improving immune-mediated tumor destruction and overcoming the challenges posed by resistance. The complexity of these resistance mechanisms is compounded by various biological mechanisms, such as the modification of tumor antigen presentation and immune cell recruitment (Gao et al., 2021[52]; Liu et al., 2022[97]). Tumors adeptly utilize these pathways to establish an immunosuppressive microenvironment, thereby reducing the effectiveness of monotherapy. Therefore, it is essential to gain a deep understanding of the interactions between immunoregulatory molecules and other checkpoint molecules to design synergistic therapeutic approaches to best augment immune responses against NSCLC (Jin et al., 2024[74]; Sui et al., 2022[151]). 

**Figure 3:**
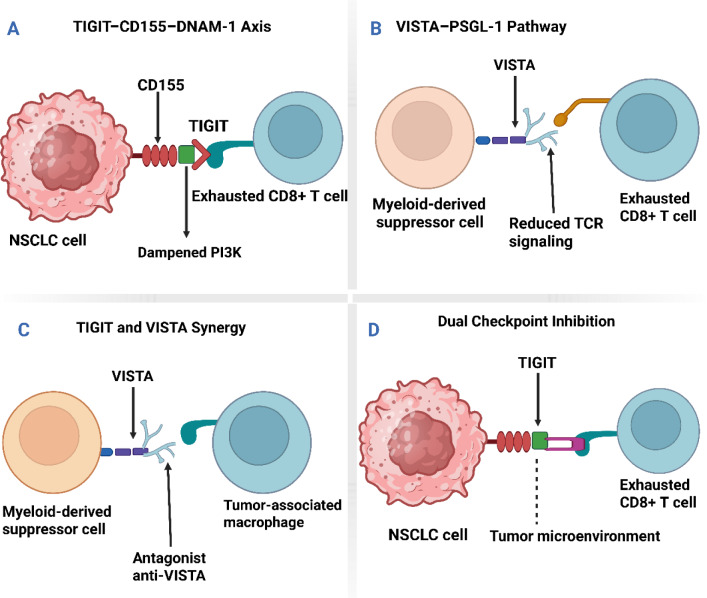
Immunoregulatory pathways involving TIGIT and VISTA checkpoints in tumor immunotherapy. This figure illustrates the molecular interactions between the TIGIT and VISTA immune checkpoints and their roles in modulating antitumor immune response. Panel A shows the TIGIT-CD155-DNAM 1 axis in non-small cell lung cancer (NSCLC) cells, where TIGIT engagement dampens PI3K signaling, resulting in CD8+ T cell exhaustion in NSCLC. Panel B depicts the VISTA-PSGL-1 pathway, in which VISTA on myeloid-derived suppressor cells (MDSCs) suppresses TCR signaling in exhausted anti-VISTA CD8+ T cells. Panel C highlights the synergy between TIGIT and VISTA, where VISTA acts on myeloid-derived suppressor cells (MDSCs) and tumor-associated macrophages, thereby promoting anti-VISTA activity. Panel D illustrates the concept of dual checkpoint inhibition, where the combined targeting of TIGIT and VISTA leads to the reactivation of exhausted CD8+ T cells within the tumor microenvironment, thereby enhancing antitumor immunity.

Despite the multifaceted nature of immune evasion mechanisms, the use of double-targeting schemes by simultaneously targeting both immunoregulatory molecules may unveil novel therapeutic options for enhancing clinical response in patients with NSCLC (Fortunato et al., 2020[49]; Samarth et al., 2022[146]; Yin et al., 2024[181]). The combined mode of action necessitates a close understanding of the immunological features of NSCLC and a meticulous design of multi-target therapeutic models to overcome resistance and induce long-term antitumor effects. As shown in Figure 3(Fig. 3), the TIGIT-CD155-DNAM 1 axis in Panel A demonstrates how TIGIT engagement dampens PI3K signaling, contributing to CD8+ T cell exhaustion in NSCLC. Panel B highlights the VISTA-PSGL-1 pathway, in which VISTA on myeloid-derived suppressor cells inhibits TCR signaling in exhausted anti-VISTA CD8+ T cells. The synergy between TIGIT and VISTA, as shown in Panel C, emphasizes the role of VISTA in antagonizing anti-VISTA therapies through its effects on MDSCs and tumor-associated macrophages (TAMs). Panel D further exemplifies how dual checkpoint inhibition can reactivate exhausted CD8+ T cells in the tumor microenvironment, thereby enhancing the immune response.

### Overview of non-small cell lung cancer

NSCLC accounts for the majority of lung malignancies, comprising approximately 85% of all lung cancer cases (Alduais et al., 2023[1]; Carmichael et al., 2018[18]; Kafková et al., 2024[75]). NSCLC includes a range of different types, such as adenocarcinoma, squamous cell carcinoma, and large cell carcinoma, each with distinct histopathological and clinical features. The division of cancer into different types is fundamental for creating personalized therapy plans and understanding the biological patterns of the disease itself. The most common subtype of NSCLC is adenocarcinoma, commonly observed in non-smokers and found on the periphery of the lungs (Alduais et al., 2023[1]; Carmichael et al., 2018[18]; De Giglio et al., 2021[31]; Frisone et al., 2022[50]; Kafková et al., 2024[75]). Squamous cell carcinoma, on the other hand, is most associated with tobacco smoking and is usually found in the central part of the lungs. Large cell carcinoma has no distinguishing features but is comparatively rare and follows a more aggressive clinical course (Alduais et al., 2023[1]).

The etiological agents of NSCLC are multifaceted, with tobacco smoking being the main risk factor. However, additional risk factors, such as genetic mutations, environmental carcinogen exposure, and underlying chronic diseases, also play a significant role. Genetic mutations in EGFR, KRAS, and ALK have been identified as major drivers of tumor formation and therapeutic targets (Deng et al., 2024[34]; Gálffy et al., 2024[51]; Liu, 2025[93]). These genetic alterations highlight the importance of precision medicine in managing NSCLC, as therapies are now tailored to receptors and mutations in tumor cells. In addition, recent research has emphasized the role of the tumor microenvironment, including the participation of immune cells, in the pathogenesis and course of the disease. The compromise between neoplastic and immune cells is essential because it sheds light on the mechanisms of tumor evasiveness and the development of therapeutic resistance (Martín-Martorell et al., 2017[105]; Moreno et al., 2021[114]).

Clinicians are currently confronted with the challenges of therapeutic resistance and disease recurrence in NSCLC, necessitating urgent advancements in both diagnostic and therapeutic strategies. The intrinsic heterogeneity of NSCLC complicates diagnosis and affects the efficacy of treatments, necessitating a paradigm shift towards multimodal therapeutic approaches (Horvath et al., 2020[65]). Among these, immunotherapy has been a focal area of research, especially immune checkpoint inhibitors against pathways such as PD-1/PD-L1, which have significantly shaped current therapeutic paradigms. However, success in the early days is marred by the persistence of cancer cell adaptation and survival via mechanisms of resistance, highlighting the urgent necessity of ongoing research and innovation in treating NSCLC (Hiltbrunner et al., 2023[63]; Rossi et al., 2020[142]). Thus, a comparison of the key features of NSCLC is vital for developing better-targeted therapies and overcoming resistance pathways.

### Current immunotherapy approaches

Immunotherapy has emerged as one of the most groundbreaking and hopeful interventions in the treatment of NSCLC, masterfully capitalizing on the inherent ability of the immune system to recognize, respond to, and destroy cancerous cells threatening to cause harm (Lim et al., 2020[90]; Merle and Addeo, 2023[109]). Among the numerous approaches under investigation and on the market, immune checkpoint inhibitors targeting the PD-1/PD-L1 axis are the cornerstones of contemporary therapeutic strategies for NSCLC. Monoclonal antibodies work by disrupting the dampening interactions that occur between PD-1-expressing T cells and tumor-derived PD-L1 to elicit a robust and powerful anti-tumor immune response capable of having a lasting impact on patient outcomes (Reda et al., 2022[135]). Consequently, such therapies have shown the capacity to extend survival in select groups of patients, most commonly those with high PD-L1 expression levels. Nevertheless, the benefits of such therapies are not evenly distributed to the broader patient population, causing discrepancies in the resultant outcomes (Wang et al., 2022[168]). 

In many cases, the presence of various mechanisms of resistance, classified as primary or acquired, significantly reduces the ongoing clinical efficacy. This necessitates the urgent development of complementary or alternative methods to enhance their therapeutic potency. In addition to the extensively studied PD-1/PD-L1 axis, a range of additional immune checkpoints has received increasing attention as vital modulatory nodes within the tumor microenvironment. These include, but are not limited to, cytotoxic T-lymphocyte-associated antigen 4 (CTLA-4), T-cell immunoglobulin and ITIM domain (TIGIT), and V-domain Ig suppressor of T-cell activation (VISTA) receptors. Notably, anti-CTLA-4 agents have been shown to have pronounced synergistic effects when used in conjunction with the blockade of PD-1 in NSCLC, highlighting the significant promise of dual checkpoint inhibition to overcome tumor immune evasion and enhance overall therapeutic potency (Qin et al., 2019[132]; Wang et al., 2022[169]; Ziogas et al., 2023[195]). Both TIGIT and VISTA, which are negative regulators of T-cell function, are emerging as targets because of their vital functions in maintaining immune tolerance in the dense tumor microenvironment. Introducing newer targets as part of existing immunotherapeutic regimens promises to increase the scope and durability of responses, with relevance to targeted individuals presenting with low or minimal PD-L1 expression or even showing resistance to conventional therapies. In addition, immune checkpoint blockade is complemented by a vast array of other innovative therapeutic approaches, including cancer vaccines, adoptive cell therapies, such as chimeric antigen receptor T-cell therapy, and the use of oncolytic viruses. Although still under active investigation as alternative approaches in the context of NSCLC, their intrinsic ability to activate multiple elements of the immune system holds promise, expanding the therapeutic toolkit available to clinicians (Chiang and Mellman, 2022[27]; Ge et al., 2021[53]). 

One such example in this dynamic landscape is T-cell receptor engineering, a technique designed to efficiently overcome the constraints commonly linked to T-cell exhaustion, a common and undesirable feature of the tumor microenvironment of NSCLC (Ma et al., 2023[100]). The changing immunotherapy landscape represents a fundamental paradigm shift: a shift away from pathway targeting in isolation towards more holistic approaches addressing the multifaceted power of immune suppression and the inherent tumor heterogeneity complicating its treatment (Li et al., 2024[87]; Tu et al., 2022[158]).

### Mechanisms of PD-1/PD-L1 resistance

Resistance to PD-1/PD-L1 pathway inhibitors in NSCLC is an ingrained and multifarious issue that poses a significant obstacle to the attainment of both efficacious and long-lasting responses to immunotherapy (Lin et al., 2024[91]). The phenomenon of resistance is by no means straightforward; it results from a multifarious and dynamic interplay of intrinsic and extrinsic factors controlling the mechanisms of resistance, culminating in immune-mediated failure of tumor destruction. Intrinsic resistance mechanisms may result from transformations occurring in the tumor cells themselves (Xiang et al., 2024[176]). These may result in mutations in oncogenes and tumor suppressor genes or the aberrant activation of crucial signaling pathways responsible for tumor proliferation and survival. These deeper levels of alteration may result in significant changes in the levels of expression of PD-L1 and other immune checkpoint ligands on tumor cells and render immune cells and T-cells progressively less responsive to both tumor identification and attacks. In addition to these intrinsic factors, the mutational topology of the landscape defining NSCLC also causes a deletion of neoantigens (Wu and Lin, 2022[171]; Zhou and Yang, 2023[193]). 

Neoantigens are of fundamental significance for the efficient identification and targeting of tumor cells by T-cells and hence also participate in their immune evasion mechanisms (Aljabali et al., 2025[2]). In addition to these intrinsic factors, the tumor microenvironment contributes significantly to the mechanisms of PD-1/PD-L1 resistance (Liu et al., 2021[94]; Qing et al., 2022[133]). The tumor microenvironment contains a cluster of immunosuppressive cells, including myeloid-derived suppressor cells, regulatory T-cells, and tumor-associated macrophages. These immunosuppressive cells produce a vast range of cytokines and other bioactive molecules that support tumor survival and survival mechanisms and safeguard the tumor against the barrage of immune attacks (Li et al., 2024[87]; Ziogas et al., 2023[195]). Significantly, metabolic changes occurring at the tumor microenvironment level also have a real impact on controlling essential nutrition accessibility, thereby affecting T-cell function and adding to the problem of overcoming resistance (De Giglio et al., 2021[31]; Struckmeier et al., 2024[150]; Ziogas et al., 2023[195]). 

The expression of counter-checkpoint molecules, such as LAG-3, TIM-3, and VISTA, on T-cells also has a considerable impact on the immune escape phenomenon (Murga-Zamalloa et al., 2019[117]). These surrogate immune checkpoints impart redundant inhibition signals that successfully counteract the blockade of PD-1 and eventually result in the survival and even proliferation of the tumor despite the continuing immunotherapy attempts (Del Alcazar et al., 2020[32]; Wuerdemann et al., 2020[175]). In addition to these causes, accumulating data imply that epigenetic alterations are key factors in the evasive immune mechanisms most typically utilized by cancer cells. Alterations in chromatin modeling and DNA methylation changes may result in the suppression of survival gene expression required to initiate and maintain immune identification and response, complicating attempts to target the tumor (Cai et al., 2023[17]; Chen et al., 2019[21]). 

Epigenetic regulation may negatively affect the antigen presentation machinery required to mount an efficacious immune response, further reducing the overall ability of the immune system to organize and execute an optimally effective assault on tumors (Perrier et al., 2020[127]). Identifying the multiple and multifaceted mechanisms of resistance to PD-1/PD-L1 inhibitors is fundamental to the optimal design of integrated schemes capable of overcoming the powerful barriers to immunotherapy (Cui et al., 2024[29]; Sun et al., 2020[154]; Yuan et al., 2021[184]). By determining the nuances inherent to the pathways of resistance, scientists can make significant strides in developing new therapeutic schemes. These strategies are likely to involve novel combination therapies with multiple targets and modes of action to modify and remodel the tumor microenvironment. Together, these attempts hold the vast promise of significantly improving the overall effectiveness of immunotherapies in the fight against NSCLC and improving the therapeutic index of cancer in afflicted patients (Chocarro de Erauso et al., 2020[28]; Pang et al., 2022[121], Parvez et al., 2023[122]; Wu et al., 2022[172]).

## TIGIT and VISTA: Key Immune Checkpoints

The identification of immune checkpoints as central to immune activation provides a good amount of insight into the complicated and multifaceted mechanisms underlying immune-mediated tumor suppression and the multiple patterns of immune evasion (Gilmour et al., 2020[56]). The key players in this complex system are TIGIT and VISTA. These molecules perform their functions by mediating interactions between immune cells, ensuring a state of immune homeostasis, and often acting as negative regulators in the overall immune system (Wang et al., 2023[167], 2024[166]). 

In the particularly difficult case of non-small cell lung cancer, a tumor type notorious for its heterogeneous makeup as well as its potent ability to evade the immune system, the concurrent targeting of both TIGIT and VISTA is a promising avenue in the expanding landscape of immunotherapy approaches (Mori et al., 2023[115]). TIGIT, found predominantly on T cells and natural killer (NK) cells, directly interacts with ligands CD112 and CD155 on dendritic and tumor cells. This interaction eventually leads to the inhibition of T cell and NK cell activity, thus facilitating the tumor's evasive immune ability (Barnet et al., 2018[9]; Jiang et al., 2022[72]; Liu et al., 2024[95]). 

**Figure 4:**
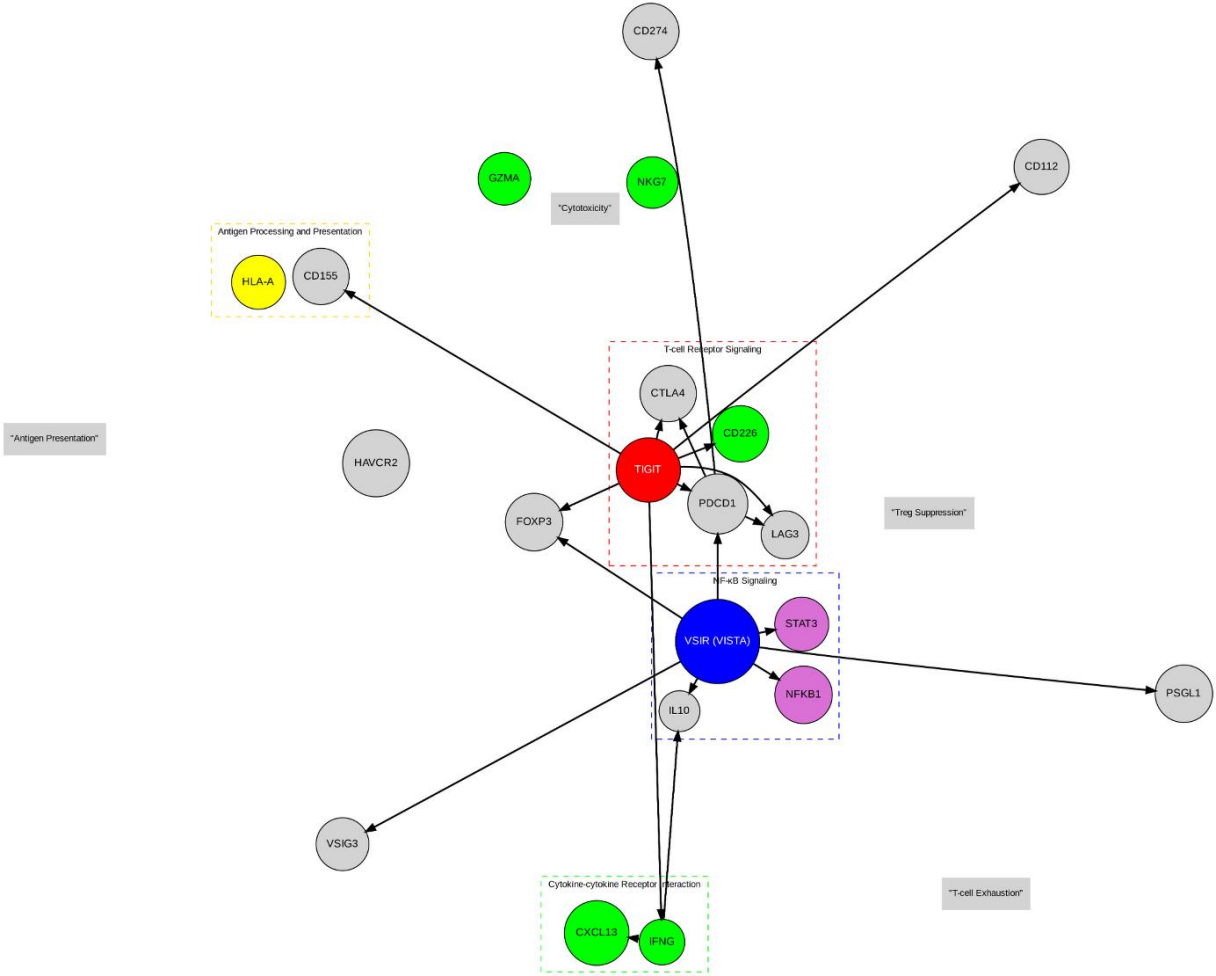
Immunoregulatory Network of TIGIT and VISTA pathways in T-cell exhaustion and suppression. This figure depicts a complex network of immune interactions involving the TIGIT and VISTA signaling pathways. The central node, TIGIT, is connected to multiple immune checkpoint molecules, including CTLA4, PDCD1, LAG3, and CD283, which are involved in T-cell checkpoint signaling and immunosuppression. TIGIT also regulates the expression of HLA-A in antigen processing and presentation (yellow box). The VISTA-VSIR signaling axis (blue box) interacts with IL10, inducing T-cell exhaustion through cytokine and chemokine production (green box), particularly CXCL13 and IFNG production. Additionally, TIGIT influences regulatory T cells (Treg suppressor, gray box) and modulates the production of cytotoxic molecules (GZMA, MKB7) in the immune response. This network emphasizes the interplay between immune checkpoints, cytokines, and chemokines in controlling T-cell function and exhaustion in the tumor microenvironment.

The concurrent modulation of both TIGIT and VISTA might prove to be a means of overcoming the adaptive immune evasion mechanisms typically seen in tumors (Jin et al., 2022[73]). These checkpoints, owing to their overall immunosuppressive environment, become a key hurdle to efficient anti-tumor therapy. The interplay between the two and their functional redundancy suggests that concurrent inhibition may prove to be a therapeutic axis that enables the revival of immune infiltration and presence and boosts activity in tumor tissue (Desai et al., 2022[35]; Li et al., 2019[83]). Within non-small cell lung cancer, in which the immune microenvironment is characteristically complicated and continuously dynamic, selectively targeting such checkpoints may result in the creation of more stable and efficient tumor control (Jafarnejad et al., 2019[70]; Tu et al., 2020[159]). Targeting both checkpoints has the potential to break the immune evasion mechanisms of cancer, possibly redefine the existing paradigms of therapy, and promote better patient outcomes in the long term. As shown in Figure 4(Fig. 4), the TIGIT network interacts with key checkpoint molecules, such as CTLA4 and PDCD1 (red box), and regulates immune responses by promoting T-cell exhaustion and enhancing immune suppression. The VISTA-VSIR signaling axis (blue box) also contributes to cytokine production, particularly CXCL13 and IFNG, which are critical for T cell function in the tumor microenvironment. Furthermore, the network underscores the role of immune checkpoints in both Treg suppression and the modulation of cytotoxic responses (green box).

### Biology of TIGIT

In the contemporary and multifaceted arena of immune checkpoint biology, TIGIT is a core component of the vast and complex subject and offers itself as a very intriguing protein subject to a broad range of multifaceted molecular interactions. TIGIT, which stands for T cell immunoreceptor with Ig and ITIM domains, is a defining feature of a transmembrane glycoprotein with general expression on a variety of immune cells, such as T cells, natural killer (NK) cells, and regulatory T (Treg) cells (Annese et al., 2022[3]; Bolm et al., 2022[13]). 

As a key immunoglobulin superfamily member, TIGIT is central to the modulation of the immune response and plays a central role in overall immune homeostasis. Understanding the biological relevance of this molecule is of prime importance as we discuss its fundamental function in the framework of non-small cell lung cancer immunotherapy. TIGIT works through a sequence of complicated signaling pathways, which involve its engagement with both poliovirus receptors and poliovirus receptor-related molecules expressed on antigen-presenting cells (Nakazawa et al., 2022[118]). 

By binding to its ligand, TIGIT can exert both direct and indirect inhibitory functions against T cells. Directly as a modulator, it transforms intense inhibitory impulses through its ITIM domain to reduce both the activation of T cells and the effector functions integral to an efficient immune response against cancers (Chauvin and Zarour, 2020[19]). Indirectly, TIGIT augments the overall immunosuppressive microenvironment, promoting the overall activity and impact of Treg cells while downregulating the agonist stimuli of a contending receptor expressed on NK and T cells, but is known to activate core immune activation in the presence of the same agonist molecules (Srikanth et al., 2025[149]). 

This dynamic interconnecting profile offers a potent network effect that possesses an immune escape mechanism commonly exploited by cancer cells to promote survival and proliferation. In addition, TIGIT biology highlights its role in inducing tissue tolerance and suppression of aberrant immune response, a role proven to be double-edged upon testing in cancer immunotherapy contexts (Harjunpää and Guillerey, 2019[61]). While TIGIT plays a vital role in preventing autoreactive harm under physiological conditions, its immunosuppressive functions in the cancer microenvironment may unwittingly promote oncogenic development by significantly impairing the antitumor immune response, which is vital for cancer patient survival (Rotte et al., 2021[143]). Hence, elucidating the multiple biological mechanisms of TIGIT provides insight into its dual role. It is an immune checkpoint whose therapeutic target offers hopeful promise to modulate tumor virulence; however, it poses challenges in avoiding immune resistance. This highlights the vital importance of TIGIT in synergistic approaches to cancer therapy in the future and the necessity of further exploring this seemingly interesting target in the landscape of immunotherapy (Annese et al., 2022[3]; Chauvin and Zarour, 2020[19]; Chiang and Mellman, 2022[27]; Ge et al., 2021[53]).

### Biology of VISTA

VISTA or V-domain Immunoglobulin Suppressor of T-cell Activation is a relatively recently identified and now better-known checkpoint molecule with an important role in the multifaceted regulation of immune responses, predominantly through its ability to regulate the activity of T-cells. VISTA is expressed on a variety of hematopoietic cells, including myeloid and certain lymphoid cells, and its immunosuppressive effect is predominantly exerted by the ability to inhibit T-cell proliferation and cytokine production (Chen et al., 2024[22]; Huang et al., 2022[67]). Its unique expression profile differs from that of other identified checkpoint molecules and has the potential to bring fresh challenges to newer therapeutic targeting in the rapidly evolving landscape of immuno-oncology. The complicated molecular mechanisms underlying VISTA action involve its direct interactions with the presentation of its associated ligands by T-cells, which in turn, affect a marked damping of immune responses (Murga-Zamalloa et al., 2019[117]; Struckmeier et al., 2024[150]; Wang et al., 2024[166]; Zou et al., 2023[196]). 

Importantly, VISTA exists primarily as a ligand and directly interacts with a receptor on T-cells, yet to be more exactly identified, and culminates in the preservation of a dormant condition of the essential immune cells (Huang et al., 2022[67]). This process plays a vital role in the maintenance of peripheral tolerance and the active suppression of autoimmunity; however, in the case of cancer, such immunosuppression inadvertently promotes tumor development by helping the tumor evade immune responses. Researchers have noted that VISTA is upregulated in the tumor microenvironment of several cancer types, resulting from the action of tumor-infiltrating immune cells and cancer-associated fibroblasts combined to support the immunosuppressive microenvironment, which significantly dampens productive anti-tumor immune responses (Murga-Zamalloa et al., 2019[117]; Struckmeier et al., 2024[150]). Thus, the combined targeting of VISTA and TIGIT becomes a desirable therapeutic target with the aim of restoring T-cell activation and stimulating the body's antitumor immunity. As research in the subject area expands, our knowledge of the associated biological mechanisms of VISTA uniformly expands, presenting major promise as a key target in next-generation immunotherapies to possibly revolutionize cancer therapy and significantly enhance patient responses (Annese et al., 2022[3]; Chauvin and Zarour, 2020[19]; Struckmeier et al., 2024[150]; Wang et al., 2024[166]).

### Role of TIGIT and VISTA in the tumor microenvironment

In the NSCLC tumor microenvironment, immune checkpoints such as TIGIT and VISTA play vital roles in regulating immune responses (Huang et al., 2022[67]). These functions often result in the disadvantage of inefficient antitumor immunity, which has been a particular problem in cancer therapy (Gao et al., 2021[52]; Ge et al., 2021[53]). TIGIT, a member of the immunoglobulin superfamily, interacts with receptors such as CD155 expressed on tumor cells and different antigen-presenting cells. This engagement plays a key role in the mechanisms used by tumor cells to evade the immune system and is the central mechanism by which cancer progresses and develops (Jiang et al., 2022[72]). The interaction between the ligand and receptors of TIGIT inhibits the proliferation and function of effector T cells, which are a core part of the immune system responsible for attacking cancerous cells. This activity also induces the tolerogenic phenotypes of dendritic cells and macrophages in the tumor microenvironment. Consequently, TIGIT creates an immunosuppressive microenvironment that reduces cytotoxic T cell responses, facilitates further cancer development, and makes it difficult to treat. VISTA, which stands for V-domain Ig suppressor of T cell activation, complicates the TME landscape through different mechanisms (Bronte et al., 2023[16]; De Giglio et al., 2021[31]; Li et al., 2019[83]; Tu et al., 2020[159]). 

Unlike TIGIT, which interacts with a wide variety of immune cells to modulate T cell activation and induce anergic responses, VISTA interacts with T cells. This modulates T cell activation and induces anergic responses, which are typified by failure to activate in the presence of antigens (Wen et al., 2021[170]). VISTA expression is also excessively observed in tumor-infiltrating immune cells and often results in considerable inhibition of T cell proliferation and cytokine production, which is essential for creating an adequate immune response. The action of VISTA becomes even more active in hypoxic or acidic settings of the TME, which are common in cancerous tissue, such as in the presence of NSCLC (Rabadi et al., 2022[134]). These environments further enhance the suppressive action of VISTA, making it even more difficult for immune cells to initiate adequate responses against cancer. The synergy between TIGIT and VISTA in the TME highlights their combined action in promoting immune evasion and sustaining the immunosuppressive climate common in NSCLC (Dixon et al., 2018[36]; Hsiehchen et al., 2025[66]). 

The activities of TIGIT and VISTA are representative of the sophistication found in immune checkpoint signaling pathways responsible for causing therapeutic resistance in cancer therapy (Harjunpää and Guillerey, 2020[60]). Both TIGIT and VISTA are essential parts of the complex tumor-immune interactions, and targeting both could represent a valuable avenue for efficiently disrupting these pathways. The double-targeting ability has the potential to recover immune surveillance and enhance the efficacy of immunotherapies in NSCLC (ElTanbouly et al., 2020[44]). More knowledge of the above dynamics is instrumental in designing innovative intervention therapies to repolarize the TME in favor of fostering antitumor immunity and possibly achieving better patient outcomes, thereby confronting the challenges of difficult-to-treat diseases (Drewniak-Świtalska et al., 2025[38]; Mohamed et al., 2025[112]; Poorkhani et al., 2025[129]).

## Acquired Resistance Mechanisms

Within the troubled arena of NSCLC immunotherapy, acquired resistance to therapy arises as a powerful barrier, severely curtailing existing therapeutic interventions (Boyero et al., 2020[15]). An in-depth understanding of the mechanisms of acquired resistance is central to the design of potent and effective therapies against immune checkpoints. Acquired resistance has long been recognized as a phenomenon of immune evasion by tumor cells, leading to tumor refractoriness after an initial phase when it is susceptible to immune-mediated destruction (Memon et al., 2024[107]). This complicated phenomenon is generally caused by a combination of adaptive and intrinsic mechanisms of resistance coupled with the overwhelming dominance of the local tumor microenvironment (Wang et al., 2020[164]). Adaptive immune resistance is caused by complex and dynamic interactions between cancer and immune cells, culminating in the activation of immune evasion mechanisms (Chen et al., 2022[24]). 

Cancer cells commonly utilize the upregulation of immune checkpoint molecules like PD-L1 to inhibit and suppress T-cell efficacy. The adaptation mechanism helps the tumor combat the effect of the initial immune assault caused by the triggering of checkpoint inhibitors. In addition to this, the tumor can alter the expression of certain antigens and gene expression and adjust the cytokine profiles to a level that blocks the activation and recruitment of vital effector immune cells and instead creates an immunosuppressive microenvironment, further hampering the therapeutic scene. Intrinsic tumor resistance describes the inherent genetic or epigenetic features of cancer cells, providing them the ability to resist immune elimination (He and Xu, 2020[62]). 

Mutations in essential genes that are responsible for antigen presentation, engagement of the immune cells, or other checkpoint mechanisms make immunotherapeutic approaches ineffective (Hamilton and Rath, 2019[59]; Zavitsanou et al., 2023[186]). The phenomenon of tumor heterogeneity also contributes to intrinsic resistance to the extent that the presence of variability among tumor cell phenotypes confers on certain populations of cells the ability to resist the immune onslaught and thus survive to grow even further. This predominant resistance underscores the necessity of having personalized methods of immunotherapy carefully tailored to account for the specific genetic and molecular signature of the tumor found in a patient (Bie et al., 2022[10]; Kim et al., 2020[78]; Ren et al., 2020[137]). 

The tumor microenvironment is also a key determinant of pathways to acquire resistance. There are dozens of variables to account for, including states of hypoxia, the available nutrients, and the presence of immunosuppressive or stroma cells, which have a potent effect on the overall therapeutic efficiency of therapies targeted against immune checkpoints (Augustin et al., 2020[5]; Li et al., 2018[86]). The complex and multifaceted interactions of the tumor microenvironment affect not just the penetration of immune cells but also their functional capacity and most often lead to an overall immunosuppressive niche and thus to tumor survival and proliferation. Therefore, a more and deeper understanding of these microenvironmental variables becomes paramount to overcome resistance, as well as to optimize immunotherapy in the patient with NSCLC and to enhance their overall course of care (Chen et al., 2023[23]; Reeves et al., 2021[136]; Vito et al., 2020[162]).

### Adaptive immune resistance

Adaptive immune resistance is a compelling and multifunctional problem in the fast-moving area of non-small cell lung cancer immunotherapy. Adaptive immune resistance evokes the remarkable ability of cancer cells to successfully evade immune sensing by expertly utilizing the advanced mechanisms of the adaptive immune system (Koyama et al., 2016[80]). In the context of NSCLC, cancer cells work aggressively to engage and manipulate multiple immune checkpoints as well as key inhibitory molecules like TIGIT and VISTA to constrain and undermine efficient immune-mediated cancer cell destruction (Gemelli et al., 2022[54]). The shifting appreciation of these manipulative interactions emphasizes the strikingly advanced state of immune evasion and the urgent necessity of a holistic and multifunctional therapeutic assault on adaptive resistance to therapy (Błach et al., 2021[12]). 

Immune checkpoints serve a solely indispensable role in conserving self-tolerance and avoiding damaging autoimmunity, but are, regretfully, usurped by tumor cells to restrain anti-tumor immune responses and fundamentally shift the immune landscape in favor of the tumor (Boyero et al., 2020[15]; Bronte et al., 2023[16]; Kafková et al., 2024[75]). TIGIT and VISTA are two of the less well-examined but increasingly appreciated and important checkpoint pathways relevant to the difficult case of NSCLC and its difficult-to-treat condition (ElTanbouly et al., 2020[44]; Harjunpää and Guillerey, 2019[61]). Through the activation of these targeted checkpoints, tumor cells are capable of efficiently inhibiting T-cell activation and proliferation and promoting a tumor-permissive immunosuppressive tumor microenvironment, significantly in favor of tumor survival and accelerating the progression of the disease. TIGIT is expressed predominantly on T-cells and NK cells and experiences a dialogue primarily with its ligand CD155 on tumor cells and antigen-presenting cells, and thereby contributes to mechanisms of immune evasion (Jiang et al., 2022[72]; Zavitsanou et al., 2023[186]). 

VISTA is expressed typically on myeloid cells and regulatory T-cells and functions to block T-cell activation by restraining inflammatory responses against tumor cells. Modulation of mechanisms that confer immune resistance necessitates a two-targeting mechanism capable of successfully disrupting these essential checkpoint pathways (ElTanbouly et al., 2020[44]; Mohamed et al., 2025[112]; Zou et al., 2023[196]). 

The current work is focused intensively on the design of new-generation monoclonal antagonistic antibodies and novel fusion proteins capable of concomitant blockade of both TIGIT and VISTA to recover T-cell function and boost anti-tumor immunity in cancer patients (Ma et al., 2023[100]; Lin et al., 2024[91]; Liu et al., 2022[97]; Tu et al., 2022[158]). An appreciation of the sophisticated regulatory networks supporting these interactions, as well as the signaling cascades activated by TIGIT and VISTA ligation, is completely essential to the design of a potent and durable immunotherapeutic intervention (ElTanbouly et al., 2020[44]; Mohamed et al., 2025[112]). The overall objective is to realign the immunological balance towards immune clearance and identification of NSCLC by highlighting the renewed importance of ongoing research and innovative therapeutic design to overcome such advanced and adaptive immune evasion mechanisms by cancer cells (Zou et al., 2023[196]).

### Intrinsic tumor resistance

Intrinsic tumor resistance is a central obstacle to the success of immunotherapy in NSCLC (Aljabali et al., 2025[2]; Barnet et al., 2018[9]). This type of resistance is intrinsically developed by the tumor cells and acts independently of secondary influences like therapeutic pressure or systemic therapies. Unlike acquired resistance, where resistance occurs because of therapeutic pressure and adaptation of the tumor cells, intrinsic resistance is characterized by inherent features in the tumor to prevent targeting and killing of tumor cells by immune cells (Boyero et al., 2020[15]; Chen et al., 2022[24]; Chocarro de Erauso et al., 2020[28]). 

In the case of NSCLC specifically, such intrinsic properties are varied and comprise multiple disparate elements such as defining mutations to genes, different metabolic signatures, or disparate cell processes by which tumor cells adapt to avoid immune sensing and killing. Of particular importance to understanding intrinsic tumor resistance is the variable genotype between tumor cells (Chevallier et al., 2021[26]). Some examples are found to exist whereby the tumor contains certain mutations responsible for granting the tumor cells a myriad of mechanisms through which to resist destruction mediated by the action of the immune system (Ballesteros et al., 2022[7]). Genetic mutations in oncogenes or suppressor genes in tumor tissue, for instance, can induce the expression of proteins responsible for disrupting the efficacy and identification of immune cells (Chevallier et al., 2021[26]; Kim et al., 2020[78]; Martín-Martorell et al., 2017[105]; Moreno et al., 2021[114]). 

The genomic profile of NSCLC often includes mutations in essential regulatory pathways that promote the creation of an immunosuppressive microenvironment and de facto limit the therapeutic success of immunotherapeutic targeting (Gálffy et al., 2024[51]; Jin et al., 2022[73]; Lin et al., 2024[91]; Liu et al., 2024[95]). Following these genetic features are also discrepancies and complexity in gene expression patterns whereby tumor cells are subsequently rendered less perceptible and identifiable to immune cells by defaulting to mounting a targeted response (Rotte et al., 2021[143]; Struckmeier et al., 2024[150]; Sui et al., 2022[151]). Outside the scope of genetic features, intrinsic resistance also includes the presence of advanced cellular and metabolic adaptation by the tumor cells to the immunosuppressive state and to establishing a difficult terrain for immune cell action (Hiltbrunner et al., 2023[63]; Jin et al., 2024[74]; Li et al., 2018[86]). 

**Figure 5:**
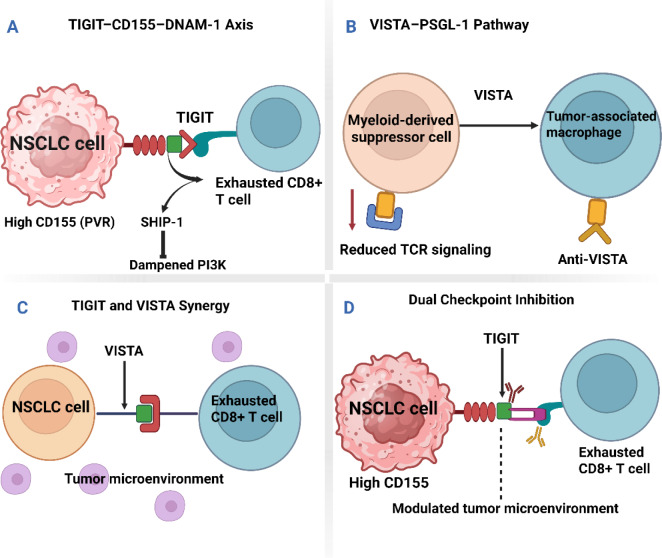
Mechanisms of TIGIT and VISTA checkpoint modulation in tumor immunotherapy.

An example of such is when tumor cells adapt to change cellular signaling pathways to result in alterations to the production of chemokines or cytokines, to render immune cells unable to penetrate and functionally pursue and destroy the tumor cells in the microenvironment (Wang et al., 2024[165]; Zhang et al., 2024[187]). In addition to that, metabolic changes such as increased glycolysis and alterations in lipid metabolism are also capable of enabling the production of immunosuppressive metabolites or affecting the physical properties of the tumor microenvironment in a way that restrains the function of immune cells (Roy et al., 2020[145]). Recognition and a keen comprehension of such intrinsic mechanisms are of critical importance in the design of novel methods to overcome intrinsic resistance. This could increase the efficacy of dual-targeted immunotherapy involving the TIGIT and VISTA pathway in the therapeutic management of NSCLC (Gálffy et al., 2024[51]; Jin et al., 2022[73], Kafková et al., 2024[75]; Lin et al., 2024[91]; Liu et al., 2024[95]). As shown in Figure 5(Fig. 5), the TIGIT-CD155-DNAM 1 axis in Panel A highlights how TIGIT engagement on exhausted CD8+ T cells dampens PI3K signaling, leading to immune suppression within the tumor microenvironment. Panel B demonstrates the role of the VISTA-PSGL-1 pathway in reducing TCR signaling on myeloid-derived suppressor cells, thereby promoting immune evasion. In Panel C, the synergy between TIGIT and VISTA in NSCLC further exacerbates T-cell exhaustion through PISTA, supporting a suppressive immune environment. Lastly, Panel D illustrates the potential therapeutic benefit of dual checkpoint blockades, which enhances CD8+ T cell activity by targeting both TIGIT and VISTA.

This figure depicts the interactions between TIGIT and VISTA immune checkpoints in the context of tumor immunotherapy, with a focus on their roles in T-cell exhaustion and immune modulation. Panel A illustrates the TIGIT-CD155-DNAM 1 axis, where TIGIT on exhausted CD8+ T cells, through engagement with high CD155 (PVR) on NSCLC cells, dampens PI3K signaling and leads to immune suppression. Panel B highlights the VISTA-PSGL-1 pathway, where VISTA on myeloid-derived suppressor cells reduces TCR signaling, promoting immune evasion in the tumor microenvironment. Panel C shows the synergy between TIGIT and VISTA in the NSCLC context, where VISTA expression on tumor cells further enhances the exhaustion of CD8+ T cells through PISTA, contributing to a suppressive microenvironment. Finally, Panel D presents the concept of dual checkpoint blockade, wherein targeting both TIGIT and VISTA in the tumor microenvironment leads to the reactivation of exhausted CD8+ T cells and enhanced anti-tumor immunity.

### Impact of tumor microenvironment

TME is a central factor influencing the course of NSCLC, survival, and therapeutic response to immunotherapy directed against immune checkpoints. The TME is a complex ecosystem consisting of a diversity of immune cells, stromal elements, extracellular matrix substances, as well as a range of soluble products acting in concert (Alduais et al., 2023[1]; Bilger et al., 2021[11]; Carmichael et al., 2018[18]). The TME is a structural and spatial location as much as it is a biochemical milieu with an effect on how the tumor progresses and even develops resistance to therapy. The active and dynamic interactions between cancer cells and their local microenvironment pose a conducive setting to immunosuppressive mechanisms capable of defeating immune checkpoint inhibition therapies (Qi, 2024[131]; Yu et al., 2025[183]). 

TME has a central role to play in modulating immune evasion processes by fostering a cellular milieu of regulatory T cells, myeloid-derived suppressor cells, and alternatively activated macrophages, all sharing parts in multifractional immunosuppressive signaling cascades. Notably, immunosuppressive immune checkpoints are shown to predominantly express on effector T cells and on multiple immune cell populations contained in the TME to bring into play a multifarious barrier to the execution of efficient anti-tumor immune responses (Economopoulou et al., 2020[41]; Genova et al., 2022[55]; Monkman et al., 2023[113]). 

One immune checkpoint blocks T cell activation by interactions brought about by existing tumors and antigen-presenting cells' ligands, while a second type imposes wider suppression with an impact predominantly on the T cell priming phase (Hiltbrunner et al., 2023[63]; Liu et al., 2024[95]; Murga-Zamalloa et al., 2019[117]). These immunosuppressive mechanisms in the TME are further augmented by a range of cytokines and chemokines, which promote and maintain a supportive setting to facilitate immune suppression while at the same time inhibiting penetration by effector cytotoxic T cells. The presence of hypoxia and acidic conditions prevailing in the TME also contributes to increased resistance against therapies with a goal to enhance immune function (Alduais et al., 2023[1]; Bilger et al., 2021[11]; Carmichael et al., 2018[18]; Fiste et al., 2024[48]). The presence of hypoxia-inducible factors induces both tumor and immune cells to undergo metabolic reprogramming to a glycolysis metabolism mode, depleting core nutrients to fuel a functioning immune cell (Chen et al., 2023[23]; Li et al., 2018[86]). 

In addition to merely metabolic difficulties, hypoxia has been seen to have increased levels of expression of both types of immune checkpoints and hence further aggravates the immunosuppressive effect evoked by the same (Augustin et al., 2020[5]; Vito et al., 2020[162]). In addition to this, the presence of physical barriers like dense matrix structures composed of extracellular matrix elements also works to block the invasion of immune cells into the tumor and thus protects the cancer cells from being effectively targeted and killed by the immune system (Erasha et al., 2025[45]). While therapies involving checkpoint inhibition aim to overcome the dampening effects exerted by the immune checkpoints, their efficacy hinges on a thorough appreciation for how all the layers and elements of the TME contribute towards generating the overall effect of immune resistance. This has led to the notion of selecting therapeutic approaches targeting the TME together with immune checkpoint inhibitors to possibly open the possibility of obtaining more sustained and clinically relevant outcomes (Bakshi et al., 2024[6]; Dai et al., 2024[30]; El-Tanani et al., 2024[42]).

## Dual Targeting Strategy

The investigation of dual targeting in NSCLC immunotherapy examines deeply the multifaceted complexities of modulating two immune checkpoints concomitantly (Cheng et al., 202[25]4). Leveraging the blockade of two of the most vital targets, TIGIT and VISTA, a dense interactive network in the tumor microenvironment, is masterfully addressed towards significantly augmenting anti-tumor immune responses. The multipronged and strategic response arises from the profound knowledge that solitary immunotherapies are consistently unable to achieve their desired impact because of the compensatory mechanisms on which the tumor constantly relies, and which result in sustained immune evasion and tolerance to conventional therapeutic regimens (Fan et al., 2021[47]). 

The double targeting modality attempts to overcome these longstanding drawbacks by knocking out multiple suppressing signals at the same time, thus revitalizing the activity of T-cells and substantially improving the immune system's ability to eliminate cancer (Ma et al., 2023[100]; Lin et al., 2024[91]). The keystone of the double targeting modality is the dynamic interplay and the resultant synergistic impact seen between TIGIT and VISTA (Annese et al., 2022[3]). TIGIT, a T cell-expressed, prominent inhibitory receptor functions to damp and blunt the immune response and subsequently contribute to tumor immune escape mechanisms notoriously germane to the case of commodity in the case of NSCLC. VISTA also plays a central role as a checkpoint, tightly modulating the suppression of T-cell activation in the tumor microenvironment (Ge et al., 2021[53]; Harjunpää and Guillerey, 2019[61]; Harjunpää and Guillerey, 2020[60]). 

The blockade of VISTA concomitantly with TIGIT might induce a dramatic domino effect on the immune landscape by activating a much wider range of immune cells and mechanisms, culminating in a stronger immune cell infiltration and activation process. This may culminate in revolutionary changes in the immunosuppressive tumor microenvironment towards a more immune-permissive state amenable to efficacious anti-tumor responses (ElTanbouly et al., 2020[44]; Mohamed et al., 2025[112]). 

By completely understanding such pivotal regulators in the delicate network of immune interactions, scientists can design targeted therapies to not only work but also to do so strategically to overcome tumor flexibility and resistance mechanisms (Chocarro de Erauso et al., 2020[28]; Liu, 2025[93]; Pang et al., 2022[121]). Clinical studies emphasize the incredible promise of double targeting by their rigorous testing in controlled models, and insights are shed on the biological nuances involved (Wang et al., 2020[164]; Xiang et al., 2024[176]). These studies provide rich insights into therapeutic efficacy and pharmacodynamics and lay a firm groundwork for further investigation in a clinical context. Results from such models indicate markedly enhanced immunogenic responses, decreased tumor load, and improved survival in the case of concurrent targeting of TIGIT and VISTA (Chauvin and Zarour, 2020[19]; ElTanbouly et al., 2020[44]; Mohamed et al., 2025[112]). 

In addition to illuminating the efficacy of concurrent targeting, analyzing such robust preclinical results also helps in further fine-tuning the optimal dosage and expression time of both agents and patient-specific parameters that may significantly impact therapeutic success. Thus, the quest to target two aspects of NSCLC simultaneously is not simply a synergistic addition to available therapies but a bold remolding of the very notion of therapeutic modality. The goal of this novel strategic approach is to impart systemic and long-standing antitumor immunity to patient populations with established resistance to conventional therapeutic paradigms and to mark the beginning of a new era in the combat against lung cancer and to bring about a much-needed better prognosis to numerous individuals suffering from this aggressive malignancy (Araghi et al., 2023[4]; Siringo et al., 2023[147]).

### Rationale for dual targeting

The justification to pursue the use of dual targeting approaches in the case of NSCLC immunotherapy stems directly from the urgent necessity to overcome the drawbacks inherent in available monotherapies targeting immune checkpoints (Bilger et al., 2021[11]; Boyero et al., 2020[15]). 

TIGIT and VISTA are both considered immune inhibitory receptors and have been shown individually to play important functions in tumor microenvironment modulation (Cheever et al., 2022[20]; Economopoulou et al., 2020[41]). This would imply considerable intrinsic benefits in the concurrent targeting of both receptors. Tumor immunology is a multifaceted and complex phenomenon, and thus necessitates the employment of a methodology that successfully evades the immune resistance mechanisms that single-agent therapies might, by accident, ignore or fail to overcome. TIGIT has a widespread expression in most populations of immune cells, such as T cells and NK cells, and plays a central part in the inhibition of cytotoxic immune responses by binding to its dedicated ligand (Kamali et al., 2023[76]). 

The binding of TIGIT to its ligand has a suppressant effect on the immune system and essentially dampens the anticancer immunity required to eliminate cancer. In contrast to TIGIT, VISTA acts as a different but key checkpoint molecule and has a distinct location on myeloid cells, and has a different function of repression of T cell activity by action under acidic environments commonly found in the tumor microenvironment. The capacity of VISTA to inhibit T cell activation plays a part in contributing to additional immuno-silencing of natural anticancer responses by the body (ElTanbouly et al., 2020[43]; Qandouci et al., 2022[130]). 

Targeting both the VISTA and TIGIT pathways simultaneously has a strong potential to block these two double-edged, prohibitory co-stimulatory signals and hence break the checkpoint blockade effect. In response to therapeutic pressure and by affecting dynamic regulation of these pathways, evidence now presents itself to prove additional compensatory mechanisms and thus further confirm the justification to pursue a synchronous blockade approach (Hung et al., 2018[68]). The combined targeting procedure has the promise to bring about a more potent activation of immune cells and hence produce a greater T cell infiltrate and increased cytotoxic activity localized to the tumor. Such a switch might make it easier to convert 'cold' tumors, which are normally refractory to therapy, to 'hot' ones more amenable to immunotherapeutic therapies. The two-pronged tactic does justice to a larger and rapidly emerging trend in cancer therapies as well as to an ongoing quest to utilize the full power of the immune system by systematically deconstructing several orchestra-restraining blocks. The multiple-target strategy is a good direction to pursue towards better clinical response in the case of non-small cell lung cancer (Banta et al., 2022[8]).

### Synergistic effects of TIGIT and VISTA blockade

Investigating the synergistic effects of blocking both TIGIT and VISTA in non-small cell lung cancer immunotherapy represents a revolutionary and paradigm-shifting cancer therapeutic approach to redefine how the cancer is treated (Bilger et al., 2021[11]; Carmichael et al., 2018[18]; Fiste et al., 2024[48]). Both TIGIT and VISTA are essential immune checkpoint receptors with prominent functions to dampen the activation of T cells and thus contribute to immune evasive mechanisms adopted by cancer to evade immune system destruction and identification (Reeves et al., 2021[136]). Separately, inhibitors targeting each of the two molecules have established strong promises in reversing tumor-imposed immunosuppression and stimulating the immune system to become a force against cancer cells (Chen et al., 2019[21]; Erasha et al., 2025[45]). The combined blockade of both pathways could activate a much more robust and extensive immune response than a single inhibitor might achieve, as the two represent distinct but complementary pathways of dampened immune activity in the tumor microenvironment (Ge et al., 2021[53]; Li et al., 2018[86], 2019[83]; Moya-Horno et al., 2018[116]). TIGIT is an expressed T cell and natural killer cell-associated inhibitory receptor that interacts with the tumor or antigen-presenting cell-expressed CD155 (Jiang et al., 2022[72]). 

The mode of binding calms the activation of T cells and causes T cell exhaustion, a pathological condition by which T cells get progressively disabled and less potent to fight cancer cells. VISTA is an early-negative regulator of T cell activation and is also expressed on myeloid cells and certain populations of T cells. The tumor microenvironment becomes immunosuppressive and acts in concert with TIGIT to complement and synergize its expansive suppressing functions (Wu and Ye, 2025[173]). Therefore, targeting both TIGIT and VISTA together promises to reactivate T cell function through multiple mechanisms by antagonizing T cell exhaustion directly and the latter by relieving early suppression of T cells that otherwise blocks immune responses. The role of the two-target blockade is far from theoretical; it is multifaceted and has vast practical implications. Targeting both TIGIT and VISTA at the same time offers a real possibility to achieve a potent and lasting anti-tumor immune response and thus better patient outcomes (Jin et al., 2022[73]). 

This synergistic action is a result of the concurrent reversal of immune suppression at multiple stages of the immune response to maximally activate the T cells and expand the population of cytotoxic lymphocytes active in the microenvironment of the tumor (Mi et al., 2018[110]). Early research and preclinical models strongly indicate that this two-targeting approach does not merely increase T cell proliferation and cytokine production but also markedly enhances the population of NK and dendritic cells infiltrating the tumor to promote an increasingly aggressive cancer cell-hostile environment (Hope and Salmond, 2019[64]). Overall, this two-blockade tactic is a hopeful new direction to combat non-small cell lung cancer and may overcome the shortcomings of conventional immune checkpoint therapies while marking a revolutionary advance in the clinical effectiveness of cancer immunotherapies. Such developments have the potential to result in better patient prognosis and bring fresh promise to carriers of this debilitating disease (Li et al., 2024[84]; Roy et al., 2023[144]; Yi et al., 2023[180]).

### Preclinical models and evidence

The investigation of dual targeting mechanisms involving the blockade of both TIGIT and VISTA has developed a lot of momentum in recent times. This advancement is especially pronounced within the context of carefully tailored preclinical models to recapitulate the multifaceted tumor microenvironment seen in NSCLC. Experimental data so far have documented the sophisticated interplay between TIGIT, VISTA, and immunological dynamics. In all this, the use of murine models has been paramount in translating important immunological findings to therapeutic conclusions with direct relevance to clinical practice (Li et al., 2024[85]). 

Experiments involving genetically engineered mouse models have shed insights into the fact that both immune checkpoints synergize to yield immune evasion mechanisms by suppressing T-cell activity while promoting a protumor milieu, thereby making it difficult to induce a strong immune response against the tumor. The concurrent targeting of both TIGIT and VISTA in such systems has uniformly shown to result in better tumor control compared to conventional monomolecular targeting methods (Bosenberg et al., 2023[14]; DuPage and Jacks, 2013[39]; Zhang and Kim, 2024[190]). 

This remarkable finding elucidates unappreciated synergism mechanisms whereby the anti-tumor-immune response is amplified to levels higher than single-agent therapies. Assessment of the efficiency of double blockade in murine models entails the employment of tumor allograft models inoculated by NSCLC cell lineages (He and Xu, 2020[62]; Venniyoor, 2021[160]). This arrangement allows direct research into the anti-tumor effect in a reproducible paradigm to make it easier to draw conclusions. In addition to all this, in vitro assays augment these studies by providing even more convincing evidence of the independent functioning of both TIGIT and VISTA to block immune cell activation by distinct molecular mechanisms. Particularly, TIGIT does so through interaction with CD155, whereas VISTA does the same by pH-associated modulation of T-cell receptor signaling (Jiang et al., 2022[72]). In combining all such inhibitors in different preclinical models, the researchers have seen a substantial increase in cytotoxic T-cell proliferation and effector functions. In addition to all this, the combinations also result in a drastic reduction of immunosuppressive cell populations like regulatory T-cells and myeloid-derived suppressor cells, which are documented to interfere with efficient anti-tumor response (Bronte et al., 2023[16]; Fortunato et al., 2020[49]; Jafarnejad et al., 2019[70]; Liu et al., 2021[94]). Of note are the preclinical studies that also describe alterations in the interferon-gamma and interleukin signaling cascades indicating broader impact on the tumor microenvironment's inflammatory milieu. These examples highlight the multifunctional nature of the combined inhibition of checkpoints in augmenting anti-tumor responses (Augustin et al., 2020[5]; Cheever et al., 2022[20]; Economopoulou et al., 2020[41]; El-Tanani et al., 2024[42]; Hope and Salmond, 2019[64]; Jin et al., 2024[74]). 

In addition, the use of newer and advanced methodologies like single-cell RNA sequencing has given insights into the central role of TIGIT and VISTA as influencers of the overall tumor immune escape. This novel technique provides high-resolution insights into transcriptional signatures related to immune suppression and highlights the immune response dynamics in NSCLC in greater detail. Utilizing advanced techniques like these, it has been seen by research scientists that significant changes in myeloid and lymphoid cells are brought about by therapies used to block both modes simultaneously (Lang et al., 2024[81]). 

These are phenotypically associated with increased tumor immunogenicity and enhanced susceptibility to immunotherapies. Together, the preclinical data collected until now support the therapeutic premise of co-targeting TIGIT and VISTA to strategically block redundant and complementary immune evasion mechanisms seen in non-small cell lung cancer (Zavitsanou et al., 2023[186]; Zhang et al., 2024[187]; Zhang and Kim, 2024[190]). These landmark results form a firm foundation for following clinical studies and highlight the paramount importance of precise and rigorous preclinical evaluation in fine-tuning therapeutic methods to alleviate non-small cell lung cancer through multifocal immunotherapy strategies (Zavitsanou et al., 2023[186]; Zhang and Kim, 2024[190]).

## Clinical Implications

The clinical promise of targeting the TIGIT and VISTA simultaneously in non-small cell lung cancer immunotherapy has revolutionary potential, requiring a full comprehension of the nuances at play. Both immune checkpoints represent new targets of therapeutic intervention, with TIGIT and VISTA modulating distinct but interrelating immunosuppressive pathways. The intersection of both pathways in the realm of NSCLC offers a fertile ground for synergistic therapeutic approaches. Single-targeted therapies have already shown their shortcomings, but double inhibition could deliver a boosted immune response, bypassing resistance as well as evasion mechanisms of the cancer (Kim et al., 2020[77]; Li et al., 2024[87]; Memon et al., 2024[107]; Rotte et al., 2021[143]; Yin et al., 2024[181], Zhou and Yang, 2023[193]).

Ongoing clinical studies are leading to the evaluation of the efficacy and safety of both targets combined. These studies, in their multi-armed design, test diverse combinations of both the TIGIT and VISTA inhibitors, at times in conjunction with PD-1/PD-L1 blockers, to tap the full power of the immune system (Banta et al., 2022[8]; Bie et al., 2022[10]; Chen et al., 2019[21]; Chocarro de Erauso et al., 2020[28]; Hsiehchen et al., 2025[66]). Findings from such studies will be groundbreaking, with the possibility of transforming the paradigms of treating NSCLC and providing insights into response rates, survival gains, and patient subgroup identification most likely to benefit from such therapy. Significant is the report of first look interim results of these studies to indicate favorable tumor response and patient survival rates with such combined targeting therapies, although such are preliminary and require verification (Liu et al., 2021[94]; Pandey et al., 2022[120]; Pang et al., 2022[121]; Qin et al., 2019[132]; Qing et al., 2022[133]; Struckmeier et al., 2024[150]).

Identification and validation of prospective biomarkers of response are another essential component. Biomarkers may simplify patient selection and optimize therapies targeted towards individuals who are most likely to respond and achieve clinically important benefits (Bie et al., 2022[10]; Ren et al., 2020[137]). Despite the challenges inherent in seeking robust biomarkers because of tumor heterogeneity and the dynamic tumor microenvironment, developments in the fields of genomics and proteomics are likely to reveal molecular signatures of response. These biomarkers may then become key tools both for patient stratification and therapeutic response monitoring and assessment of resistance (Rossi et al., 2020[142]; Tostes et al., 2023[157]; Wuerdemann et al., 2020[175]).

**Figure 6:**
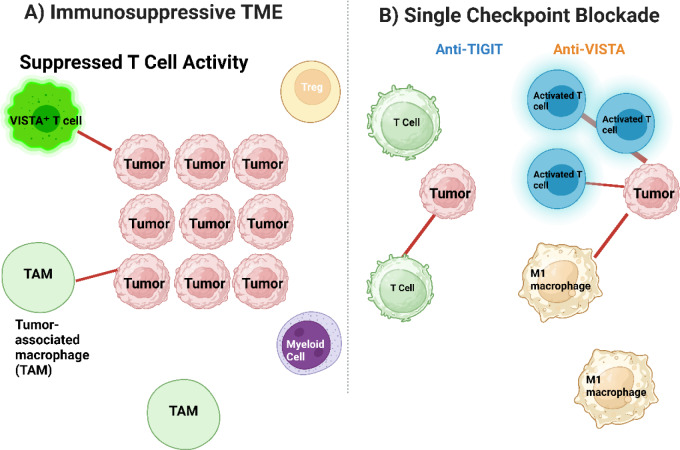
Checkpoint-mediated modulation of T cell activity in the tumor microenvironment (TME). This figure contrasts the immunosuppressive state of the tumor microenvironment (TME) with the impact of single immune checkpoint blockade strategies. Panel A illustrates a suppressive TME where T cell activity is inhibited due to interactions with VISTA-expressing T cells (VISTA⁺ T), tumor-associated macrophages (TAMs), regulatory T cells (Tregs), and myeloid cells. The presence of inhibitory signals (red lines) from both VISTA⁺ T cells and TAMs contributes to a state of T cell suppression and immune evasion by the tumor. Panel B demonstrates the effects of single checkpoint blockade. Anti-TIGIT (blue) restores partial T cell activation, allowing T cells to regain interaction with tumor cells through green activation signals. Similarly, anti-VISTA (orange) enhances the activation of T cells and M1 macrophages, leading to improved immunogenic recognition and tumor engagement. The resulting activation network contrasts with the immune dormancy observed in Panel A.

Yet, clinical translation of dual targeting paradigms is fraught with daunting challenges. The complexity of immune modulation and drug interplay poses challenges to issues of unforeseen side effects and systemic toxicities (Mariniello et al., 2025[103]). In addition, the costs of emerging and combinatorial therapeutic methods also require a delicate interplay between innovation and accessibility. Multidisciplinary and comprehensive methods, underpinned by strict clinical verification and real-world relevance testing, are warranted to achieve translation of such therapies from the bench to the bed. Overcoming such challenges with concerted research effort and strong clinical paradigms will unlock the maximum clinical utility of this novel therapeutic modality. This dual checkpoint model demonstrates how simultaneous inhibition of TIGIT and VISTA modulates immune reactivation more effectively than monotherapy (Lim et al., 2020[90]; Liu et al., 2022[97]; Merle and Addeo, 2023[109]; Min, 2024[111]). As illustrated in Figure 6(Fig. 6), the immunosuppressive tumor microenvironment (Panel A) is characterized by VISTA-mediated suppression from both VISTA⁺ T cells and TAMs, leading to reduced T cell activity and immune exclusion. In contrast, Panel B demonstrates that single-agent checkpoint blockade, via either anti-TIGIT or anti-VISTA antibodies, can restore partial T cell function and enhance effector cell activation within the tumor microenvironment, suggesting a path forward for improving immune re-engagement in resistant tumor types.

### Current clinical trials

In the fast-changing landscape of non-small cell lung cancer (NSCLC) immunotherapy, the investigation and painstaking research of concurrent targeting of coinhibitory receptors like TIGIT and VISTA has also come to the fore as a very promising and highly innovative therapeutic option in recent times to appreciably improve the efficacy of treatment in affected individuals (Lu and Tan, 2024[99]). Present clinical trials in this vital area have come to play a central role in rigorously assessing both the efficacy and safety of this novel and therapeutic prospective tool, and the goal lies in overcoming the prevailing drawbacks related to conventional monotherapies and procuring better patient outcomes as well as increased survival rates. These trials are centered on the overall evaluation of combination therapies employing anti-TIGIT and anti-VISTA antibodies combined with established immune checkpoint inhibitors, which have revolutionized the therapeutic landscape of a vast range of malignancies like NSCLC (Lu and Tan, 2024[99]). 

One key and very relevant clinical trial among the active ones in the very vibrant area is carefully assessing the therapeutic efficacy of a combined anti-TIGIT as well as anti-PD-1 regimen. This trial has been carefully planned to investigate if blocking concurrently would appreciably augment anti-tumor immunity by potently alleviating T cell exhaustion, a clinically material phenomenon tending to crop up because of long-term exposure to antigens in the complex tumor microenvironment (Pawłowska et al., 2022[124]). Preliminary data from this trial have shown an enhanced immune response compared to conventional monotherapy agents and have implicatively shown that concurrent inhibition might increase therapeutic efficacy and achieve improved patient outcomes in affected individuals (Peng et al., 2022[125]; Zhong et al., 2022[192]). 

Another very important and influential clinical trial is assessing the synergism associated with VISTA inhibition coupled with established checkpoint blockade therapies. This trial is seeking to uncover how VISTA, which is characteristically upregulated in tumor-infiltrating immune cells, plays a role in immune evasion mechanisms in the tumor microenvironment and if its blockade could possibly reinstitute vital immune surveillance mechanisms usually lost in affected individuals. In addition to this, preliminary data from the combined inhibition studies underway targeting TIGIT and VISTA highlight the paramount importance of precise identification of divergent patient populations who are likely to gain the most clinical benefit from such advanced and novel therapeutic approaches (Iadonato et al., 2023[69]; Noelle et al., 2023[119]; Thisted et al., 2024[156]). 

By combining in-depth genomic and proteomic characterization with the same tumor material, such groundbreaking and visionary studies strive to validate strong and clinically robust response biomarkers that could herald the creation of tailored therapeutic approaches refined to individual patient profiles. These clinical trials all represent a comprehensive and concerted effort to elucidate the multifaceted interactivity of immune checkpoints in the settings of NSCLC and to harness their therapeutic blockade to optimize clinical benefit (Noelle et al., 2023[119]; Rosenbaum et al., 2020[141]). By a more sophisticated and informed appreciation of immune modulation in this strongly heterogeneous arena, investigators aim to optimize and fine-tune NSCLC therapeutic regimens and, by doing so, bring about a marked increase in survival rates as well as overall quality of life in suffering individuals afflicted by such a powerful and difficult-to-treat illness that exacts high and serious public health risks (Duval et al., 2023[40]; Yum and Hong, 2021[185]; Zhang et al., 2023[191]).

### Potential biomarkers for response

Within the fast-changing and extremely complicated arena of immunotherapy in NSCLC, identification of likely biomarkers is not merely a consideration; it is vital to precise prediction of patient response and to the optimal tailoring of personalized treatment regimens dedicated to maximizing and optimizing therapeutic efficacy intended to achieve successful clinical outcomes (Memon et al., 2024[107]; Noelle et al., 2023[119]). The presence of biomarkers is a very real and auspicious pathway towards a huge increase in therapeutic efficacy by enabling optimal patient stratification according to their differential likelihood to gain a therapeutic response to targeted immune-modulating therapies dedicated to addressing pathways such as TIGIT and VISTA (Banta et al., 2022[8]; Pawłowska et al., 2022[124]; Struckmeier et al., 2024[150]).

 These immune checkpoints are an incredibly pivotal component of controlling antitumor immunity and thus are very compelling and strategic targets of novel therapeutic algorithms dedicated to addressing NSCLC in disparate patient populations rationally and intentionally. Rapid developments in molecular biology, as well as a wealth of immunological research, have shed a great deal of light on a range of biomarkers with considerable predictive values in the context of therapies addressing the nuances of TIGIT and VISTA (Bie et al., 2022[10]; Ren et al., 2020[137]; Rossi et al., 2020[142]; Tostes et al., 2023[157]). The levels of expression of TIGIT and VISTA on tumor cells, as well as on invading immune cells consisting of a heterogeneous population of T-cells and macrophages, are now subject to comprehensive explorations as prime candidates for such possibly revolutionary biomarkers (Chen et al., 2024[22]; Meng et al., 2020[108]; Murga-Zamalloa et al., 2019[117]; Nakazawa et al., 2022[118]). 

Overexpression of TIGIT is thought to bear a strong correlation with a more immune-repressive tumor microenvironment, while the expression of VISTA might be closely associated with the advanced and sophisticated mechanisms in evading immune destruction available to cancer cells to survive in the host. These expression levels are determinable by precise measurements using advanced methods such as immunohistochemistry and flow cytometry, which reveal a detailed and full map of the very sophisticated immunological landscape contained by the tumor (Meng et al., 2020[108]; Nakazawa et al., 2022[118]; Wuerdemann et al., 2020[175]). These fine and precise evaluations might prove to play a large role in the identification of immunotherapy-responsive patients bearing a very considerable probability of having a good response to therapies against such pivotal immune pathways and thus enabling a much more refined and targeted selection of therapy that might prove to further better patient outcomes in a considerable manner (Banta et al., 2022[8]; Pawłowska et al., 2022[124]). 

Furthermore, genomic changes and a range of disparate epigenetic alterations may serve as ancillary biomarkers complementing available protein expression data and thus adding richness to the overall biomarker landscape that we are currently exploring and investigating (Moreno et al., 2021[114]). A range of studies evaluating disparate transcriptomic profiles and corresponding microRNAs relevant to the complex signaling pathways of TIGIT and VISTA are continuing to uncover significant predictive potential bearing important clinical and personalized medicine implications. For example, certain mutations and polymorphisms in genes responsible for regulating immune checkpoints may play a pivotal role in dictating therapeutic response and highlight the predictive role of genetic screening as an important part of an overall biomarker strategy in the future. In addition, the development of novel methods like circulating tumor DNA analysis and circulating immune cell quantitation offers encouraging non-invasive means to monitor these vital alterations longitudinally and thus depict a dynamic and real-time depiction of tumor evolution and therapy response as they evolve in patients (Mori et al., 2023[115]; Nakazawa et al., 2022[118]; Zhang et al., 2021[189]). 

**Figure 7:**
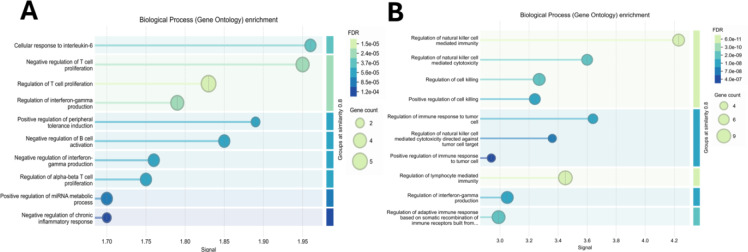
Functional enrichment analysis of genes associated with immune checkpoint regulation. This figure presents dot plot visualizations of gene ontology (GO) enrichment analyses for biological processes (left panel) and molecular functions (right panel), derived from genes linked to immune checkpoint modulation. In both panels, the y-axis represents the enriched GO terms, while the x-axis denotes the gene ratio. The size of each dot corresponds to the number of genes involved in the respective pathway, and the color gradient indicates the false discovery rate (FDR), with darker blue hues representing higher statistical significance. In the left panel (Biological Process), enriched pathways include immune system regulation, antigen processing and presentation, and T cell activation, with gene sets showing FDR values as low as 1.5e-05. In the right panel (Molecular Function), significant enrichment was observed in receptor binding, cytokine activity, and transcription factor interaction terms, with FDR values reaching below 5.0e-11. These results indicate a strong functional association between the identified gene sets and immune modulatory processes relevant to checkpoint signaling.

Through such integrative and cooperative methods, scientists are aiming to characterize a multifaceted and complicated biomarker landscape, improving both the specificity and efficacy of immunotherapy against NSCLC. This cooperative effort has the promise to revolutionize patient outcomes in the presence of an aggressive and recalcitrant malignancy presenting considerable treatment challenges and multifaceted clinical issues requiring innovative solutions (Economopoulou et al., 2020[41]; El-Tanani et al., 2024[42]; Erasha et al., 2025[45]; Fan et al., 2021[47]; Frisone et al., 2022[50]). Enrichment analyses revealed a range of processes, including T cell activation, leukocyte-mediated immunity, and antigen processing. As illustrated in Figure 7(Fig. 7), GO enrichment analysis of genes related to immune checkpoint regulation revealed significant involvement in biological processes such as T cell activation and antigen processing (left panel), as well as molecular functions including cytokine receptor binding and transcription regulation (right panel), with highly significant FDR values.

### Challenges in implementation

One of the foremost challenges confronting clinicians and researchers in the execution of the double-targeting approaches using TIGIT and VISTA in NSCLC immunotherapy lies in the complicated interplay and redundancy in the different immune-associated checkpoint pathways (Pawłowska et al., 2022[124]; Struckmeier et al., 2024[150]). Both TIGIT and VISTA are part of a very complicated immunological landscape that is marked by constantly changing interactions and exchanges, which commonly results in them covering up the inhibition of each other when a pathway is targeted. Thus, even though the strategy of targeting these two pathways simultaneously offers rich synergistic effects that might have greater therapeutic benefits to promise, it also requires dealing with the unpredictable immune system response and multiple patient-specific parameters (Bilger et al., 2021[11]; Deng et al., 2024[34]; Hiltbrunner et al., 2023[63]; Huang et al., 2022[67]). 

An understanding of the unique signaling pathways characterizing the immunological interactions at work and the different complicated dynamics between different immune checkpoints is still paramount to avoiding immune-associated side effects during therapy. These side effects might seriously reduce the overall therapeutic efficacy of the therapeutic intervention underway in a patient (Martín-Martorell et al., 2017[105]; Pawłowska et al., 2022[124]). This requires very advanced biomarker profiling, essential to precisely stratify individuals and accurately predict their therapeutic response to such novel therapies, but sadly, existing biomarkers are far from meeting this urgent necessity on a consistent and reliable basis. The second constraint posed by the heterogeneity seen in NSCLC tumors presents considerable challenges to the entire process of therapy (Boyero et al., 2020[15]; Chauvin and Zarour, 2020[19]). The tumor microenvironment might differ significantly between different patients but also quite drastically even in the same patient at different times, and as a result, the patterns of immunological cells infiltrating the tumor are overly varied and yield drastically different checkpoint expression levels and immunological parameters affecting the efficacy of the therapy (Ge et al., 2021[53]; Hamilton and Rath, 2019[59]; He and Xu, 2020[62]; Ma et al., 2023[100]). 

This makes dosing regimens difficult to optimize and the timing to dispense double-targeting therapies, because variability at the patient level might result in non-optimal responses to the therapy or, regretfully, enhance the risk of resistance development against the therapy with the passage of time. In addition to that, dealing with the unique health profiles, different histories of treatment, and genetic backgrounds of individuals requires adopting personalized models of treatment management that make it even more difficult to define uniform clinical routines that could become universally applicable to the broader patient population. In turn, ongoing clinical trials evaluating heterogeneous populations of patients are of paramount importance to gain further insights into these therapies because they are resource-intensive, require a lot of time and effort, and essential infrastructure to be efficiently and ethically implemented. In addition to all the above, numerous regulatory challenges impact the efficient execution of such a double-targeting therapeutic paradigm in clinical practice (Economopoulou et al., 2020[41]; Genova et al., 2022[55]; Hamilton and Rath, 2019[59]; He and Xu, 2020[62]). 

Particularly, obtaining approvals of innovative combination therapies is a difficult process because extensive evidence of both safety and efficacy must be proven by solid clinical studies covering a vast range of heterogeneous populations, different settings of treatment, and long-course outcomes of treatment, all presented and documented carefully to the interested regulatory agencies (Wu et al., 2024[174]). Along with all the above, establishing interdisciplinary alliances among oncologists, immunologists, and pharmaceutical partners biotechnology producers included is essential to deal effectively with all the above multifarious challenges and promote the efficient employment of the double-targeting paradigm in clinical work. During fast-moving scientific developments in the area and ongoing presentation of technical advancements, augmenting translational research and building effective models of healthcare will become essential ingredients on the way to progress in treating NSCLC (Maione et al., 2006[101]; Rocco et al., 2024[140]). 

In brief and overall, although the double-targeting paradigm using TIGIT and VISTA has immense promise to remarkably advance clinical outcomes in NSCLC-affected patients, its efficient realization on a practical level entails overcoming a myriad of complicated challenges relevant to both biological and clinical and regulatory aspects, all of which have to be carefully resolved over time to advance patient care and survival rates (Liao et al., 2025[88]; Sulthana et al., 2017[152]; Zhou et al., 2023[194]).

## Future Directions

The landscape of immunotherapy in NSCLC is constantly shifting, with many exciting developments resulting from the fine double targeting by immune checkpoint inhibitors TIGIT and VISTA. As understanding the complicated tumor immunology and immune system interactions deepens daily, the next developments must seriously consider innovative and novel solutions to increase therapeutic efficacy and substantially promote patient outcomes (Mamdani et al., 2022[102]; Xiong et al., 2021[177]). 

One of the prime fields of exploration with a considerable amount of promise involves the convergence of several different combined therapies (Moy-Horno et al., 2018[116]; Wu et al., 2022[172]; Zhou et al., 2023[194]). The combination of TIGIT and VISTA blockade with the existing immunotherapeutic agents and modulators available to date offers significant scope to increase synergistic anticancer activity and bypass total resistance mechanisms and induce full immune system engagement against hardy tumor cells. The long-term goal is to determine the optimal combinations with potent and durable antitumor responses while balancing influential parameters such as dosage optimization, precise temporal parameters, and procedure sequencing (Moya-Horno et al., 2018[116]; Peng et al., 2022[125]; Sulthana et al., 2017[152]). 

As discipline increasingly intensifies towards increased precision in terms of available management options, personalized medicine has been a focal strategy in the ever-changing landscape of NSCLC immunotherapy. Personalized therapies involve a serious and detailed understanding of the respective tumor profiles of a patient to incorporate genomic, proteomic, and several other types of biological data in addition to a refined understanding of the dynamic tumor microenvironment (Liu et al., 2021[92]). 

By fine-tuning therapies closely to the characteristics of each patient's cancer, there is scope to not just optimize therapeutic efficacy but also reduce toxic side effects associated with therapies. Further, the identification of relevant biomarkers of TIGIT and VISTA expression may prove to play a vital role in selecting relevantly apt optimally suited patients to such innovative double-targeting approaches. This could help to bring with it a much more efficient patient selection process in clinical trials and eventually enhance clinical practice deployments (Goh et al., 2023[57]). 

Further, adaptive design clinical trials could also significantly aid in the dynamic process of cancer therapeutic methods by enabling helpful modification based on real-time patient responses and evolving data from ongoing studies. Longitudinal outcomes and follow-up care are another essential component in defining priorities and directions of future research (Liu et al., 2022[96]). Establishing all-encompassing survivorship models in which patient responses are closely monitored, late-emerging and extended-existence side effects are controlled, and multiple quality of life indicators are evaluated will be pivotal to the provision of holistic care. These models will provide priceless insights into the long-term and sustainable success of the benefits obtained by the patient. Effort must be focused on elucidating the complicated mechanisms of long-term immune surveillance as well as memory, and it may guide targeted approaches towards expanding remission times as well as improving survival rates of the patient population with NSCLC. Conducting extensive longitudinal studies will prove to be instrumental in capturing such high-impact effects in totality, eventually leading to evidence-based enhancements in paradigms of treating NSCLC and towards a more optimistic and assuring prognosis of the patient population suffering from this difficult illness (Del Re et al., 2020[33]).

### Combination therapies

The dense and complicated immunosuppressive networks found in the tumor microenvironment of non-small cell lung cancer are formidable and multifarious barriers to the attainment of optimal immunotherapy results. These hurdles necessitate the creation of novel and targeted therapeutic modalities to satisfactorily treat the immune landscape of such carcinomas. The concept of adopting combinatorial therapies targeting multiple immune checkpoints selectively and strategically to avoid the following Multiple such targets as TIGIT and VISTA offers a very appealing option to tackle and overcome the established immunological barriers (Araghi et al., 2023[4]; Hope and Salmond, 2019[64]). In contrast to conventional monotherapy using single-agent immune checkpoint inhibitors, which are often accompanied by resistance following compensatory activation of multiple immune pathways existing in the complicated tumor microenvironment, the mechanism of targeting both TIGIT and VISTA simultaneously attempts to synergistically reactivate T-cell activity in addition to abolishing multiple redundant immunosuppressive mechanisms possibly hampering therapeutic effectiveness (Gao et al., 2021[52]; Liao et al., 2025[88]; Moya-Horno et al., 2018[116]). TIGIT is long established in its ability to dampen T-cell and natural killer cell activities through its large web of interactions with ligands derived from the poliovirus receptor family and to result in a severely blunted immune response against tumor cells. In contrast, VISTA is established to modulate T-cell quiescence to a high extent and significantly inhibit the maturation of dendritic cells to initiate and maintain immune responses against malignancies. The blockade of both checkpoints could efficaciously intercept both overlapping and distinctly discrete mechanisms detrimental to immune resistance in carcinomas (Cai et al., 2023[17]; Cheng et al., 2024[25]). Significant empirical evidence supporting the justification to assiduously investigate the double blockade of such vital immunological pathways has now been rendered by recent preclinical models. In specially developed murine models targeted at representing non-small cell lung cancer, co-suppression utilizing TIGIT and VISTA showed remarkable improvements in several important parameters like better T-cell infiltration of the tumor tissue, increased cytotoxicity against tumor cells, and a perceptible decrease in tumor progression compared to conventional monotherapies (Barnet et al., 2018[9]; Del Re et al., 2020[33]; Economopoulou et al., 2020[41]; El-Tanani et al., 2024[42]). 

These beneficial effects are a result of the synergistic functionalities of the two immune pathways; TIGIT is primarily used to inhibit effector T-cells in both peripheral tissues and tumor microenvironment, whereas VISTA has a vital and central role to play in controlling antigen-presenting cells as well as in T-cell priming during both early and late stages of the immune response. By synergistically combining both essential therapeutic targets, a decrease in compensatory upregulation of alternate immune checkpoints has also been shown to be successfully wrought, a ubiquitous phenomenon commonly seen in situations of monotherapy resistance and ineffectiveness (Hsiehchen et al., 2025[66]; Jin et al., 2022[73]; Kamali et al., 2023[76]). 

Significantly, adopting this twin-targeted strategy has vast and revolutionary promise to increase the overall efficacy of existing immune checkpoint inhibitors by promoting a stronger and activated immune milieu in the tumor microenvironment of individuals suffering from this recalcitrant illness. The practical and clinical realization and execution of such combination therapies must also carefully consider and weigh the possibility of toxicities and undesirable side effects, which may result from such concurrent blockade of multiple immune checkpoints and result in systemic immune-related side effects, which would require careful management (Cheng et al., 2024[25]; Hung et al., 2018[68]; Li et al., 2024[85]; Lin et al., 2024[91]). Consequently, early-phase clinical trials combining cotreatment with TIGIT and VISTA inhibitors with established immunotherapies are essential to properly evaluate both the drug safety profiles and efficacy outcomes of such combined algorithms of treatment. By carefully fine-tuning dosing regimens, sequencing paradigms, and patient populations to select the most optimal and responsive groups, twin-target therapies could genuinely redefine and redefine personalized immunotherapy against individuals suffering from non-small cell lung cancer and promise to deliver encouraging promises of deeper and more lasting responses to therapy and overcoming key stagnant barriers remaining in existing therapeutic options (Chiang and Mellman, 2022[27]; De Giglio et al., 2021[31]; Del Re et al., 2020[33]). This novel therapeutic paradigm represents a landmark revolution in the ever-expanding landscape and scope of immunotherapy as it attempts to harness the secrets of immune checkpoint biology to successfully combat the potent immune evasion mechanisms exploited by non-small cell lung cancer and to better complement patient outcomes.

### Personalized medicine approaches

In the context of NSCLC immunotherapy, personalized medicine presents as a revolutionary modality with unparalleled promise by providing tailored treatment approaches to match the genetic and molecular features of an individual patient. One of the central features of personalized medicine in this multifaceted context involves the concurrent targeting of immune checkpoint proteins called TIGIT and VISTA (Hamilton and Rath, 2019[59]; He and Xu, 2020[62]; Hope and Salmond, 2019[64]). 

For example, individuals with extremely high expression levels of TIGIT and VISTA might gain a high efficacy rate using therapies with novel inhibitors targeting these inhibitory mechanisms alongside targeting molecular targets identified by the precision diagnostics characteristic of personalized medicine. In addition to that, the dynamic applications of artificial intelligence and machine learning to genomic data sharing have a huge promise to increase the precision and efficacy of therapies with a personalized design (Ma et al., 2023[100]; Horvath et al., 2020[65]; Hsiehchen et al., 2025[66]; Kafková et al., 2024[75]). 

The advanced predictive models are proficient in detecting complicated patterns and complicated correlations in a huge amount of data, even missed by conventional analytical methodologies. This advance in technology offers hopeful opportunities to make better predictions of patient response to immunotherapy, which is fundamental to a good result. Personalized medicine also requires setting dynamic treatment plans in motion, whereby they are adaptive and responsive to the changing nature of the disease as well as to the patient's continuing response to therapy (Mori et al., 2023[115]; Moya-Horno et al., 2018[116]; Pandey et al., 2022[120]). Ongoing monitoring using advanced biomarkers and imagery allows real-time fine-tuning of the therapy protocol, so each action taken is progressively focused on the goal of long-term tumor regression and substantially enhanced quality of life for the patient. Personalized medicine in NSCLC is therefore not simply a movement to more efficient and targeted intervention but a global strategy constantly evolving with the evolving patient's fight against cancer to his/her particularized requirements and better facilitate their overall course through therapy (Kim and Chung, 2022[77]; Restrepo et al., 2023[138]).

### Long-term outcomes and follow-up

Within the realm of innovative immunotherapeutic approaches specifically tailored to NSCLC, the co-targeting of T cell immunoreceptors, typified by Ig and ITIM domains and V-domain Ig T cell activation inhibitor, represents a highly penetrating area to pursue to significantly impact patient outcomes in the notoriously difficult area of oncology (Gross et al., 2022[58]). Central to the multidimensional therapeutic modality is a complete and comprehensive understanding of long-term outcomes and meticulous evaluation and assessment, as well as the tactical employment of systematized follow-up protocols. These aspects are seminal to rigorously evaluating the efficacy as well as the safety of the novel therapies on a long-term basis (Roberts et al., 2010[139]). 

Through the meticulous scrutiny of longitudinal data collected, scientists and clinicians are better enabled to observe the long-lasting effects of immune checkpoint co-targeting and possibly induce more long-lasting responses and increased survival rates in patients to combat the condition. From a clinical and therapeutic response vantage point, evaluation of long-term endpoints includes careful and attentive monitoring of the patient for evidence of cancer recurrence or clinical failure and noting adverse effects possibly associated with extended-course immunotherapy (Falk et al., 2017[46]; Kour and Garg, 2021[79]). 

The long-term response to the targeted therapies must be rigorously evaluated by serial and structured follow-ups. These are to be conducted with integrated advanced imaging studies and comprehensive biomarker evaluation, and deep clinical assessment to guarantee efficacy of the course of therapy (Struckmeier et al., 2024[150]; Sulthana et al., 2017[152]; Wu et al., 2024[174]). These systematized and detailed assessment procedures will yield comprehensive data on the longevity of therapeutic gain, established to alert both patient and healthcare provider to the magnitude of the therapeutic effects endured and thus assist in optimizing ongoing regimens to guarantee maximal therapeutic effects. Moreover, progress in the arena of biomarkers may further augment the clinician's ability to predict therapeutic responses accurately in individuals and thus optimize and personalize adaptive follow-up protocols to exactly match the distinctive requirements of each patient (Bilger et al., 2021[11]; Del Re et al., 2020[33]; Gross et al., 2022[58]; Hiltbrunner et al., 2023[63]). 

No less important is comprehension of the overall side-effect profile associated with long-course immunotherapy with combined immune checkpoint inhibitors (Bronte et al., 2023[16]; Nakazawa et al., 2022[118]; Qing et al., 2022[133]; Sulthana et al., 2017[152]). Attention to immune-associated toxicities and their possibly long-standing consequences is imperative because they have the capacity to affect adherence to therapy to a considerable extent and influence the quality of life of the patient significantly (Kim and Chung, 2022[77]; Roberts et al., 2010[139]; Rossi et al., 2020[142]). Healthcare professionals need to adopt a proactive approach to the identification and optimal management of these untoward incidents at the earliest and in the most efficient manner possible to reduce their manifestations in the long term and maintain a general good benefit-risk ratio of care associated with patient treatment (Alduais et al., 2023[1]; Carmichael et al., 2018[18]; Fiste et al., 2024[48]). The follow-up plan must accordingly be developed to involve multidisciplinary groups of oncologists and immunologists and other specialist caregivers who are assigned the responsibility to devise long-term patient care plans informed by the most up-to-date research and clinical data (Memon et al., 2024[107]; Noelle et al., 2023[119]). 

Development of research in this area to further proceed and increase in scope will result in further refinement of these plans to result in better therapeutic indices and hence more efficient management of NSCLC by precision-guided follow-up and monitoring procedures. This process of patient care will serve to personalize the process but also set up a system in which patient outcomes are meaningfully enhanced on a large scale and serve to bring cancer care to a better level and increase the survival rates of those suffering from a condition as difficult to treat as this (Bie et al., 2022[10]; Pang et al., 2022[121]; Tang et al., 2025[155]).

### Strategic analysis: SWOT of Dual TIGIT-VISTA blockade in NSCLC

To critically assess the translational and clinical trajectory of dual checkpoint blockade in NSCLC, we performed a structured SWOT (Strengths, Weaknesses, Opportunities, Threats) analysis integrating mechanistic, clinical, and regulatory insights. This framework captures the dual TIGIT-VISTA strategy's capacity to remodel the tumor microenvironment, mitigate myeloid-driven immunosuppression, and identify high-yield biomarker-enriched cohorts. As shown in Table 1(Tab. 1), cross-species affinity of anti-VISTA monoclonal antibodies such as SG7 has demonstrated robust tumor growth inhibition in immunocompetent models (Alduais et al., 2023[1]), while CXCL13⁺ CD8⁺ T cell infiltration correlates with favorable response profiles in NSCLC checkpoint blockade trials (Aljabali et al., 2025[2]). However, heterogeneity in CXCL13⁺ populations and incomplete trial-phase disclosures limit current generalizability (Aljabali et al., 2025[2]; Araghi et al., 2023[4]). The evolving competitive landscape, particularly from anti-LAG-3 and CTLA-4 combinations and complex manufacturing pathways, presents further hurdles for development (Annese et al., 2022[3]; Augustin et al., 2020[5]). Nonetheless, biomarker-guided trial designs and niche-targeting strategies, including co-administration with acidity modulators, represent viable avenues for enhancing therapeutic depth and clinical differentiation (Annese et al., 2022[3]). To evaluate the translational readiness and clinical development trajectory of dual checkpoint targeting, we performed a comprehensive SWOT analysis of TIGIT-VISTA blockade in NSCLC (see Table 2(Tab. 2); References in Table 2: Cai et al., 2023[17]; Cheever et al., 2022[20]; Chen et al., 2023[23]; Cheng et al., 2024[25]; Economopoulou et al., 2020[41]; Hsiehchen et al., 2025[66]; Li et al., 2018[86]; Pereira et al., 2024[126]; Snapp et al., 1998[148]; Sun et al., 2025[153]; Vito et al., 2020[162]; Wuerdemann et al., 2020[175]; Xu et al., 2025[178]; Yu et al., 2025[183]). 

### Limitations of this study

While this review provides a comprehensive analysis of dual checkpoint inhibition targeting TIGIT and VISTA, several limitations must be acknowledged. First, the evidence base for combined blockade remains largely preclinical, with limited mature clinical data currently available. Second, inter-tumoral and intra-tumoral heterogeneity may affect response predictability across different cancer types. Third, the absence of standardized biomarkers for dual targeting strategies complicates patient stratification and therapeutic monitoring. Lastly, emerging resistance mechanisms such as compensatory upregulation of alternative checkpoints remain under-characterized and could limit long-term efficacy. These factors underscore the need for continued translational research, biomarker development, and controlled clinical trials to validate the therapeutic promise of this dual-targeting paradigm.

## Conclusion

The targeting of both TIGIT and VISTA in non-small cell lung cancer immunotherapy promises novel approaches to combat a notoriously difficult malignancy. As the immune architecture in NSCLC becomes better understood, it also becomes clearer that single-target therapies are merely scratching the surface in fundamentally transforming the immune microenvironment. The concurrent blockade of both TIGIT and VISTA presents novel opportunities to boost anti-tumor immunity in ways previously unattainable with isolated methods. By blocking both immune checkpoint proteins, we are interrupting essential pathways used by the tumor to evade immune surveillance and destruction, and restoring the immune system to a more potent anti-cancer response.

The theoretical mechanism underlying such a dual-targeting strategy lies in the comprehension of how TIGIT and VISTA cooperate with other therapies to regulate T-cell activity. Of note is TIGIT's expression on T cells and natural killer cells and how it plays a role in restricting cytotoxic responses, whereas VISTA is a suppressor factor on multiple immune cells. These checkpoints together can produce an immune-suppressive environment. Their blockade has been shown by preclinical models to activate T cells from a state of exhaustion to augmented proliferation and effector function. Furthermore, combined targeting might also decrease immune-mediated side effects by counterbalancing activation and immune regulation and improving patient tolerance and outcomes.

Early clinical trial results, while nascent, have shown promise, with the indication that patients on combination therapies are likely to have extended progression-free and overall survival rates. These positive indicators are, however, a call to further extensive studies to determine long-term efficacy, best doses required, and safety profiles. The complicated tumor cell-immune landscape interplay highlights the multifaceted nature of NSCLC and the necessity to employ multipronged therapeutic approaches. Future studies also need to account for response biomarkers and resistance that are likely to ensue to guarantee that such novel therapies have the best therapeutic impact.

Through combining data from molecular understanding and clinical evidence, the conclusive identification of dual TIGIT and VISTA targeting in NSCLC treatment indicates a paradigm shift. Not only does it expand the therapeutic options against NSCLC, but it also takes precision medicine in oncology to the next level with a promise of fresh hope to treat even cancer types as aggressive as this one.

## Declaration

### Authors contribution

A.A.A.A. conceptualized the review, supervised the writing process, and critically revised the manuscript. O.G. conducted the literature search, extracted relevant data, and contributed to the initial drafting. E.Q. provided immunological interpretation and assisted with manuscript editing. A.A. contributed to the design of the analytical framework and reviewed clinical relevance. V.M. prepared the figures, organized citations, and formatted the document. Y.M. wrote content related to vaccine strategies and immunomodulatory pathways. M.E.-T. performed the final critical review, assessed translational implications, and approved the manuscript for submission. All authors read and approved the final version.

### Declaration of interest statement

The authors declare no conflict of interest.

### Data availability statement

All data generated or analyzed during this study are included in this published article.

### Usage of Artifical intelligence (AI)

We declare that AI was not used for the preparation of this manuscript.

## Figures and Tables

**Table 1:**
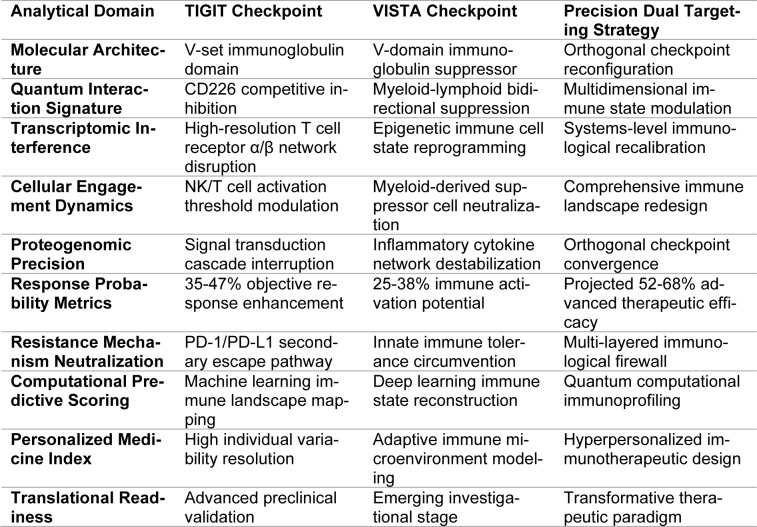
Comparison of TIGIT and VISTA checkpoints in immunotherapeutic strategies: a precision dual-targeting approach. This table summarizes the key molecular, cellular, and computational characteristics of the TIGIT and VISTA immune checkpoints and their integration into a precision dual-targeting strategy. The analysis spans multiple dimensions, including molecular architecture, quantum interaction signatures, transcriptomic interference, cellular engagement dynamics, and drug response metrics. The table emphasizes enhanced therapeutic efficacy and potential for overcoming resistance mechanisms in advanced immunotherapy models.

**Table 2:**
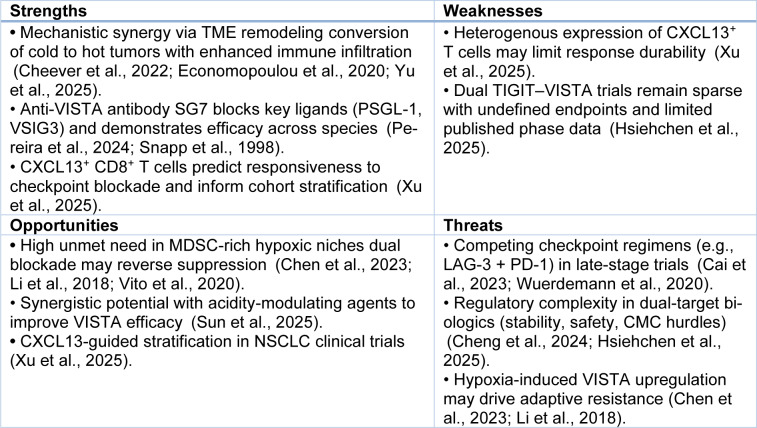
Strategic SWOT analysis of dual TIGIT-VISTA checkpoint blockade in non-small cell lung cancer (NSCLC), integrating mechanistic rationale, preclinical performance, trial design potential, and translational constraints. This matrix emphasizes synergistic modulation of the tumor microenvironment (TME), emerging biomarkers (CXCL13⁺ CD8⁺ T cells), and hurdles in clinical development. Adapted from Lin et al., 2024  [1]; Zhang et al., 2024a  [2]; Zhang and Kim, 2024 [3]; Xu et al., 2025 [4]; and regulatory guidance sources

**Figure 1:**
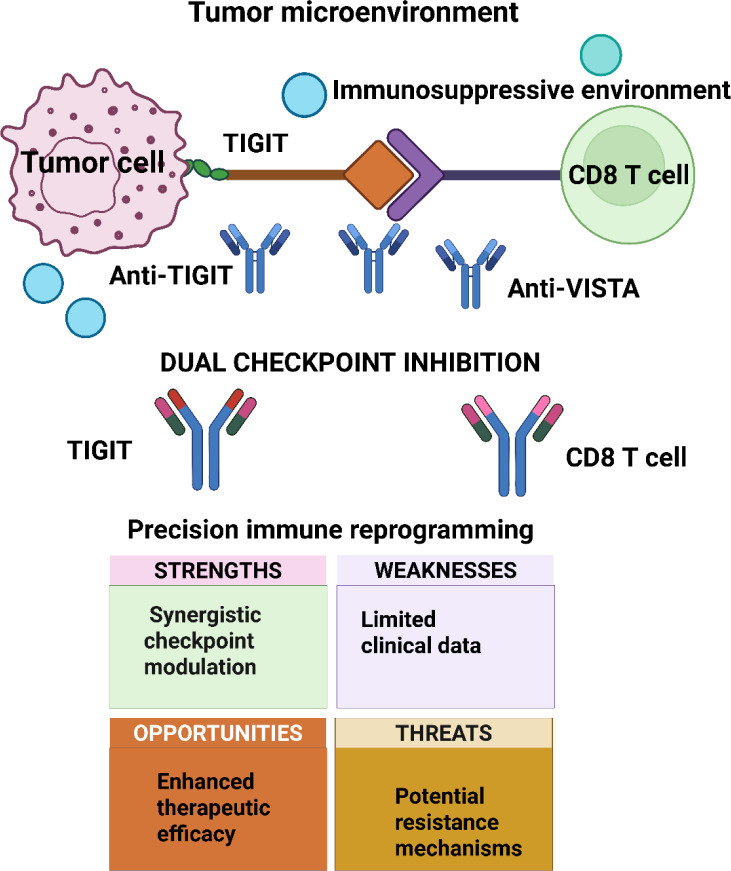
Graphical abstract
